# Regulation of inorganic polyphosphate is required for proper vacuolar proteolysis in fission yeast

**DOI:** 10.1016/j.jbc.2021.100891

**Published:** 2021-06-18

**Authors:** Naoya Sawada, Shiori Ueno, Kojiro Takeda

**Affiliations:** 1 Graduate School of Natural Science, Konan University, Kobe, Hyogo, Japan; 2 Institute for Integrative Neurobiology, Konan University, Kobe, Hyogo, Japan

**Keywords:** autophagy, vacuole, polyphosphate, life span, cell cycle, fission yeast, EDAM, electron-dense, amorphous material, EMM2, Edinburgh Minimal Medium 2, InsP, inositol polyphosphate, NLA, nitrogen limitation adaptation, PBS, phosphate-buffered saline, PMSF, phenylmethylsulfonyl fluoride, polyP, polyphosphate, ROS, reactive oxygen species, TBS, tris-buffered saline, TORC1, target of rapamycin complex 1

## Abstract

Regulation of cellular proliferation and quiescence is a central issue in biology that has been studied using model unicellular eukaryotes, such as the fission yeast *Schizosaccharomyces pombe*. We previously reported that the ubiquitin/proteasome pathway and autophagy are essential to maintain quiescence induced by nitrogen deprivation in *S. pombe*; however, specific ubiquitin ligases that maintain quiescence are not fully understood. Here we investigated the SPX-RING-type ubiquitin ligase Pqr1, identified as required for quiescence in a genetic screen. Pqr1 is found to be crucial for vacuolar proteolysis, the final step of autophagy, through proper regulation of phosphate and its polymer polyphosphate. Pqr1 restricts phosphate uptake into the cell through ubiquitination and subsequent degradation of phosphate transporters on plasma membranes. We hypothesized that Pqr1 may act as the central regulator for phosphate control in *S. pombe*, through the function of the SPX domain involved in phosphate sensing. Deletion of *pqr1*^*+*^ resulted in hyperaccumulation of intracellular phosphate and polyphosphate and in improper autophagy-dependent proteolysis under conditions of nitrogen starvation. Polyphosphate hyperaccumulation in *pqr1*^*+*^-deficient cells was mediated by the polyphosphate synthase VTC complex in vacuoles. Simultaneous deletion of VTC complex subunits rescued Pqr1 mutant phenotypes, including defects in proteolysis and loss of viability during quiescence. We conclude that excess polyphosphate may interfere with proteolysis in vacuoles by mechanisms that as yet remain unknown. The present results demonstrate a connection between polyphosphate metabolism and vacuolar functions for proper autophagy-dependent proteolysis, and we propose that polyphosphate homeostasis contributes to maintenance of cellular viability during quiescence.

The transition between cellular proliferation states, *i.e.*, active mitosis or cellular quiescence (G_0_ phase), is finely controlled in response to intra- and/or extracellular conditions, such as the availability of growth factors (animal cells) or nutrients (yeasts), and remains a central issue in biology (1, 2). Failure to control entry into or exit from quiescence may lead to cancer. Moreover, maintenance of differentiated postmitotic cells in quiescence is strongly relevant to pathology of diseases such as neural degeneration. Therefore, regulatory mechanisms governing transitions into and out of quiescence have medical importance.

Like budding yeast (3), the fission yeast, *Schizosaccharomyces pombe*, has served as an excellent model to study cellular quiescence, because the transition between vegetative proliferation and quiescence can easily be controlled experimentally by adding or removing a nitrogen source in the media (4). Under nitrogen deprivation, after dividing twice without cell growth, *S. pombe* cells exit vegetative proliferation and enter quiescence. These cells remain metabolically active in quiescence for at least 2 months without growth or division (5, 6). Upon replenishment of nitrogen source, they exit quiescence and resume vegetative proliferation. By exploiting these characteristics of *S. pombe*, comprehensive genetic approaches using mutant libraries have been employed to identify genetic mechanisms governing quiescence (1).

Various genes and cellular pathways have been identified as requisite for quiescence (5, 7, 8, 9). Among them, the ubiquitin/proteasome system is important to sustain viability of quiescent *S. pombe* cells (9). Proteasome dysfunction in quiescent cells results in significant autophagy-mediated degradation of mitochondria and accumulation of reactive oxygen species (ROS). These findings implicate proteasomes and autophagy in governing quality control and abundance of mitochondria, respectively, although detailed mechanisms have yet to be identified (9). Which ubiquitin ligases are responsible for viability in quiescence and governing mitochondria? Identification of responsible factors may reveal basic principles underlying the pathology of neurodegenerative diseases resulting from abnormalities of these processes.

Recently, it was shown that polyphosphate (polyP), a polymer of inorganic phosphate (10, 11), participates in proteostasis (12). In *Escherichia coli*, polyP acts as a protein chaperone and prevents protein aggregation under harsh conditions, such as oxidative stress (13). PolyP also modifies amyloidogenesis, which is involved in protein-folding diseases such as Alzheimer's and Parkinson's diseases (14, 15). In mice and rats, the brain contains abundant polyP and its level reportedly declines with age (16, 17). PolyP is also thought to be engaged in starvation responses of unicellular organisms (18, 19, 20, 21). In *E. coli*, polyP contributes to amino acid recycling to activate LON protease, which degrades ribosomal proteins and supplies amino acids during nitrogen starvation (19). In budding yeast, inactivation of polyphosphatases (polyphosphate hydrolytic enzymes) has a lethal effect on the stationary phase, although molecular mechanisms of lethality are not understood (20).

In this study, we investigated an uncharacterized, putative ubiquitin ligase, designated Pqr1, which was originally identified during genetic screening, to find fission yeast mutants of ubiquitin ligase showing defects in viability and/or mitochondrial maintenance (*e.g.*, mitochondrial degradation, mitochondrial morphology, etc.) during quiescence induced by nitrogen source withdrawal. In our analyses, Pqr1 was not a specific regulator of mitochondrial degradation, but it proved indispensable for general autophagic proteolysis induced by nitrogen starvation. The specific function of Pqr1 is to restrict phosphate uptake by downregulating phosphate transporters on plasma membranes and to sustain appropriate levels of cellular phosphate, especially polyphosphate. Hyperaccumulation of polyphosphate, invoked by Pqr1 dysfunction, led to defective vacuolar proteolysis, causing incomplete autophagy-dependent proteolysis and shortened life span during quiescence.

## Results

### Identification of Pqr1

In our previous study, we reported that proteasomes and autophagy are important for mitochondrial maintenance and viability during quiescence (9). In proteasome-deficient fission yeast cells during quiescence induced by nitrogen deprivation, autophagy-mediated destruction of mitochondria occurs, which may suppress accumulation of ROS to preserve cellular viability. However, the ubiquitin ligases involved are unknown, so we attempted to find mutants defective in sustaining viability and/or mitochondria during quiescence.

As a pilot study, 70 ubiquitin ligase mutants (gene deletion or temperature-sensitive) were investigated. We found a gene deletion mutant of a putative ubiquitin ligase encoded by SPAC6B12.07c, which showed loss of viability in quiescence/G_0_ phase induced by nitrogen deprivation, abnormal degradation of mitochondria by autophagy, and fragmented mitochondria. However, this study revealed that the ubiquitin ligase is important for proteolysis dependent on starvation-induced autophagy, but that it is not specific to mitochondrial autophagy (mitophagy (22, 23)) as shown later. We designated the ubiquitin ligase encoded by SPAC6B12.07c as Pqr1 (proteolysis factor that quiescence requires) and sought to determine the mechanism by which this ubiquitin ligase guarantees autophagic protein recycling and sustains cellular viability under nitrogen starvation.

### Pqr1 is required for viability during quiescence induced by nitrogen starvation

**Figure 1:**
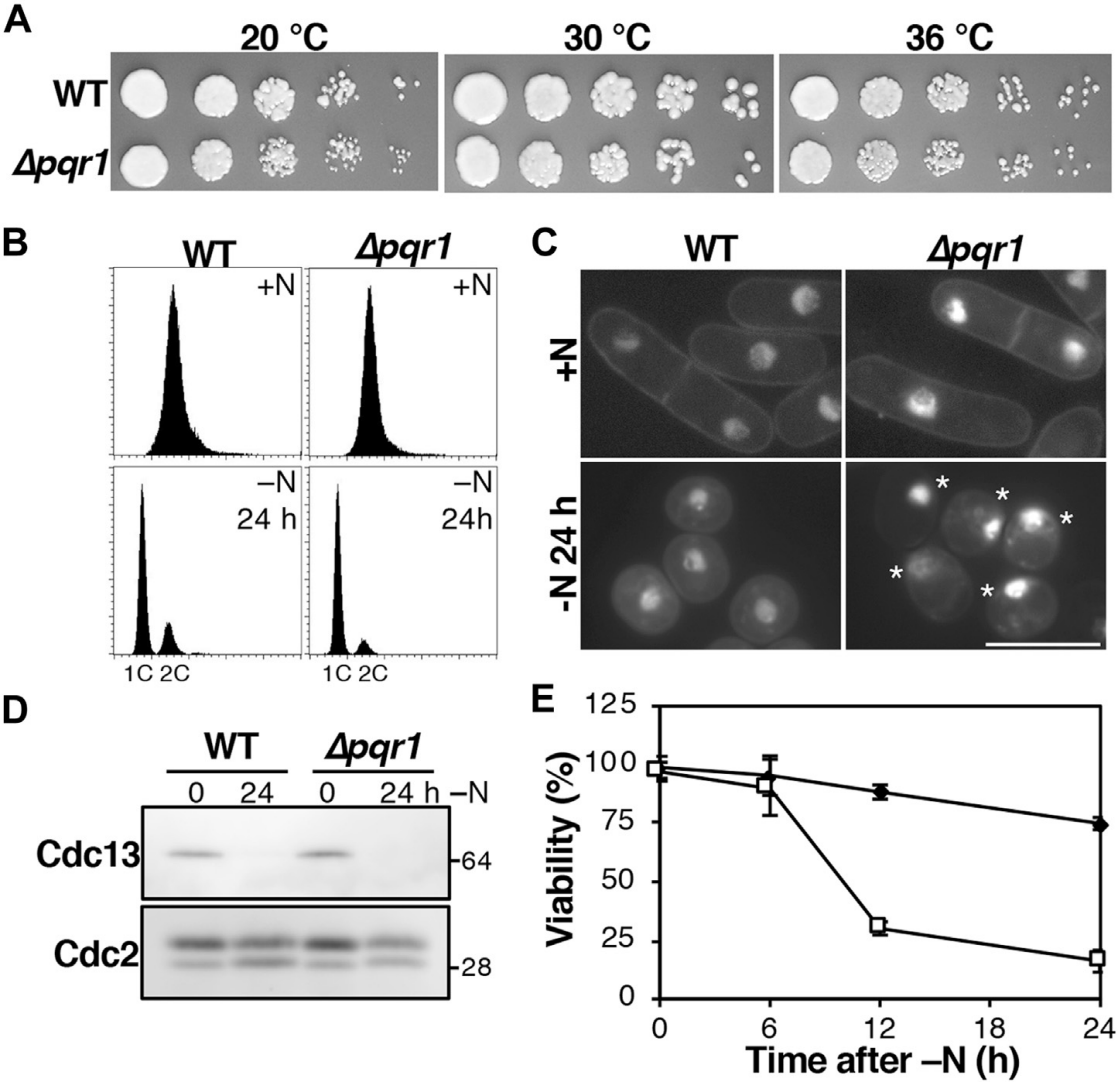
**Pqr1 is required to maintain viability during −N.***A*, *Δpqr1* and WT cells grew on the synthetic medium EMM2 at 20, 30 and 36 °C. *B*, both WT and *Δpqr1* arrested the cell cycle with 1C DNA content 24 h after −N. *C*, cellular morphologies of indicated strains were obtained by staining DNA and cell walls with DAPI. *Asterisks* indicate *Δpqr1* cells with abnormally positioned nuclei. Bar, 10 μm. *D*, Cdc13, the mitotic cyclin, was degraded 24 h after −N in WT and *Δpqr1*. *E*, viabilities of WT and *Δpqr1* after −N. Experiments were repeated 3×, and means and SDs are presented.

Cellular functions of Pqr1 were investigated by means of a deletion mutant of the *pqr1*^*+*^ gene (*Δpqr1*). The proliferation rate of *Δpqr1* mutants on synthetic Edinburgh Minimal Medium 2 (EMM2) was almost indistinguishable from that of the wild-type strain 972 (WT), while *Δpqr1* grew slightly slower at lower temperature (Fig. 1*A*). Then, we examined phenotypes of *Δpqr1* under nitrogen starvation (−N). Upon nitrogen withdrawal, effected by changing the medium from EMM2 (containing 93.5 mM NH_4_Cl as the sole nitrogen source) to EMM2 without NH_4_Cl (EMM2–N), cell number increased (Fig. S1), and ploidy and morphology of *Δpqr1* cells were analyzed and compared with those of WT (Fig. 1, *B* and *C*). *Δpqr1* cells divided twice, arrested with 1C DNA content (before S phase), and displayed reduced cell size, as did WT cells at 24 h under −N. Although nuclei are usually located in the center of the cell, in *Δpqr1* mutants, nuclei were located at the cell periphery (Fig. 1*C*). Mitotic cyclin Cdc13 was decreased in *Δpqr1*, as well as in WT (Fig. 1*D*) (5). These results suggest that Pqr1 is dispensable for entering quiescence induced by −N. However, we found that Δ*pqr1* mutants lose viability after −N (Fig. 1*E*). Taken together, we concluded that Pqr1 is not required for vegetative proliferation or for entry into quiescence, but that it is required for maintenance of viability during quiescence induced by −N.

### Pqr1 is important for autophagy-dependent degradation in vacuoles under −N

**Figure 2:**
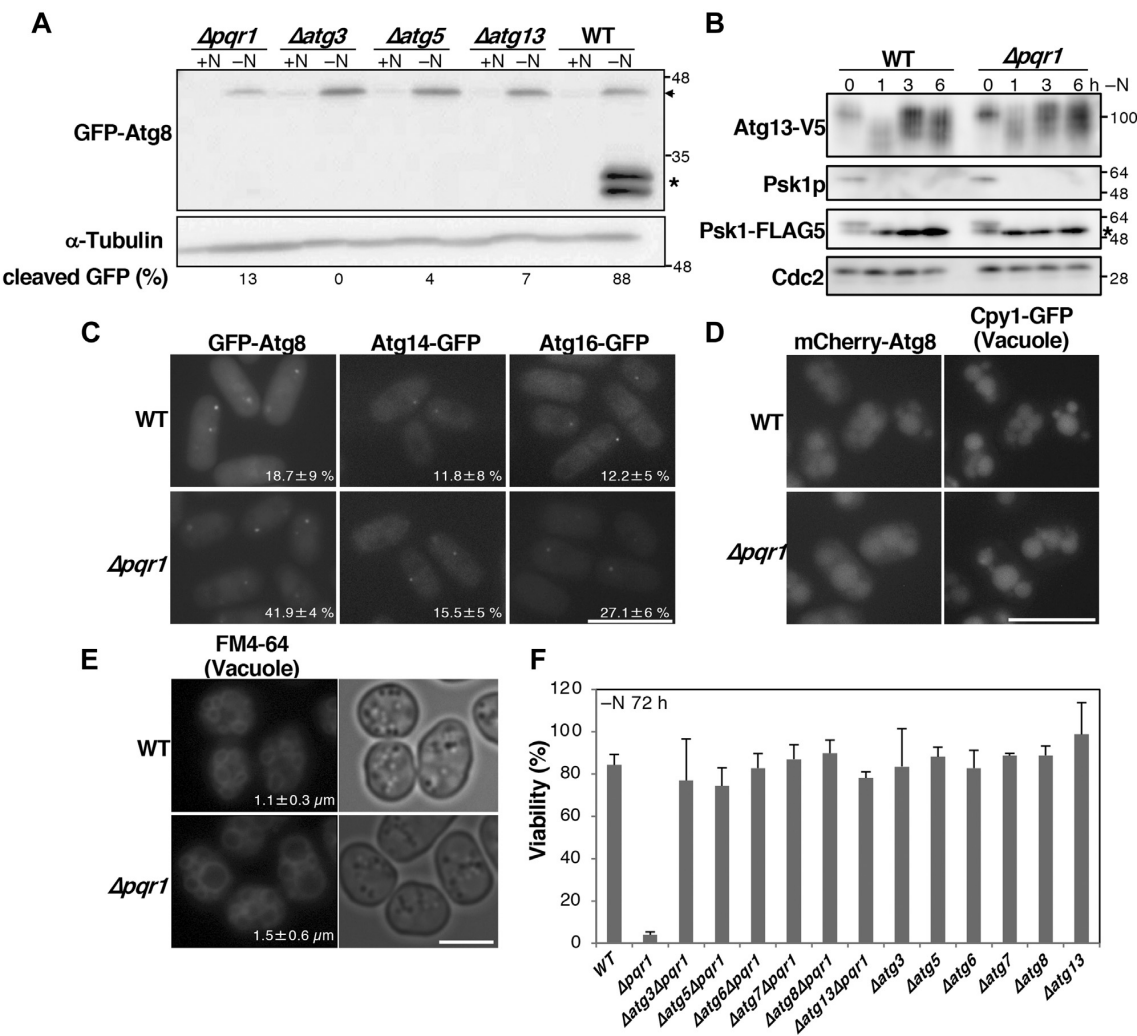
**Pqr1 is required for autophagy-dependent proteolysis in vacuoles.***A*, GFP-Atg8 (*arrow*) was cleaved and free GFP (*asterisk*) was detected in WT, but hardly seen in *Δpqr1* or *Δat*g mutants 24 h after −N. Ratios (%) of cleaved-GFP (cleaved GFP/[cleaved-GFP + GFP-Atg8]) are shown. *B*, dephosphorylation of TORC1 targets, Atg13 and Psk1 after −N. Psk1p indicates immunoblot using anti-phospho-Psk1 antibody. The *asterisk* indicates the phosphorylated upper band. *C*, PAS formation in WT and *Δpqr1*. Ratios (%) of cells with PAS are represented (means and SDs). Bar, 10 μm. *D*, mCherry-Atg8 was incorporated into vacuoles in both WT and *Δpqr1* after −N. Cpy1 (Carboxypeptidase Y)-GFP was used as a marker of vacuolar lumens. Bar, 10 μm. *E*, vacuolar membranes were stained with FM4-64. Means and SDs of vacuolar diameter are represented. Vacuolar diameters in *Δpqr1* were significantly longer than in WT (*p* < 0.01). Bar, 5 μm. *F*, viabilities of indicated strains at 72 h after −N. The viability loss of *Δpqr1* was suppressed by simultaneous deletion of *atg* genes. Experiments were repeated 3×, and means and SDs are presented.

We next investigated whether Pqr1 has specific functions in mitochondrial degradation. If Pqr1 is specific to mitochondria and is not involved in general autophagy, starvation-induced autophagy may not be affected overall. In WT cells, Atg8 protein on autophagosomes is normally degraded in vacuoles. To determine whether this occurs in *Δpqr1* cells, the GFP-Atg8 method was adopted (24, 25). In WT under −N conditions, autophagy was active, as confirmed by detecting cleaved-GFP by immunoblot (Fig. 2*A*). In autophagy-deficient mutants, namely *Δatg3*, *Δatg5*, and *Δatg13*, and in *Δpqr1*, cleaved-GFP was not detected. If Pqr1 is specific to mitophagy and does not affect nonselective autophagy, cleaved GFP should be detected. This result suggests that in *Δpqr1* cells, autophagy-dependent proteolysis may be compromised, rather than mitophagy.

We then asked which steps in the autophagy cascade are affected in *Δpqr1*. First, we examined the activity of target of rapamycin complex 1 (TORC1), a serine/threonine kinase that controls autophagy (26, 27, 28). Upon −N, TORC1 is immediately inactivated, resulting in dephosphorylation of Atg13, which triggers an autophagy cascade (29). In *S. pombe*, TORC1 activity is monitored by phosphorylation states of the S6 kinase ortholog (Psk1) and Atg13 (30, 31). To detect Psk1 and Atg13 by immunoblot, endogenous genes of *psk1*^*+*^ and *atg13*^*+*^ were C-terminally tagged with FLAG5 or V5. The anti-phospho-S6 (T389) antibody reportedly recognizes Psk1 phosphorylated by TORC1. Immunoblot analyses showed that Psk1, recognized by anti-phospho-S6 kinase (T389) antibody, disappeared within an hour after −N in both WT and *Δpqr1*, suggesting that TORC1 was inactivated (Fig. 2*B*, Psk1p). Dephosphorylation of Psk1 was also confirmed by immunoblot using anti-FLAG antibody. The phosphorylated upper band (asterisk in Fig. 2*B*), corresponding to the band detected with the anti-phospho-S6 antibody, disappeared within an hour. Consistently, the electrophoretic mobility of Atg13-V5 increased within an hour after −N in WT and *Δpqr1*, suggesting that dephosphorylation of Atg13-V5 normally occurs in *Δpqr1* cells. Hence, we conclude that Pqr1 is not required for sensing nitrogen starvation and inactivation of TORC1.

Then, we examined formation of pre-autophagosomal structures (PAS) in *Δpqr1*. Atg proteins involved in autophagosome nucleation have been reported to form foci, called PAS, after activation of autophagy (25, 32). Localization of Atg8, Atg14, and Atg16 was visualized using GFP (Fig. 2*C*). The gene for GFP was C-terminally fused to endogenous *atg14*^*+*^ or *atg16*^+^ and was fused N-terminally to *atg8*^*+*^ using a plasmid vector for chromosomal integration. The expression construct of GFP-Atg8 controlled by the promoter of *atg8*^*+*^ was integrated into the *leu1*^*+*^ locus on chromosome II. We then investigated localization of these Atg proteins in WT and *Δpqr1* 6 h after −N and found that all Atg proteins formed PAS, even in *Δpqr1* (Fig. 2*C*).

To complete autophagy-dependent proteolysis, autophagosome-vacuole fusion is necessary, so we examined it in WT and *Δpqr1*. Because GFP fluorescence is sensitive to acidic environments, as in vacuolar lumens, we created a strain expressing mCherry-Atg8. mCherry fluorescence is less affected by pH. If autophagosomes are formed and engulfed by vacuoles, mCherry-Atg8 on autophagosome membranes should be transported into vacuoles, where protease-resistant mCherry should accumulate inside vacuoles. WT and *Δpqr1* cells expressing mCherry-Atg8 were shifted to EMM2–N for 8 h, and we observed that the mCherry signal accumulated in vacuoles in both strains (Fig. 2*D*), suggesting that autophagosome-vacuolar fusion occurs in the absence of Pqr1. These results indicate that nitrogen-sensing, autophagy activation, and autophagosome-vacuole fusion occur normally in *Δpqr1* as well as in WT; however, degradation of engulfed autophagosomes may be impaired in *Δpqr1*.

### Vacuolar abnormalities in *Δpqr1*

To better understand vacuoles in *Δpqr1*, we visualized vacuoles with FM4-64, a fluorescent dye that stains vacuolar membranes (Fig. 2*E*). We observed that vacuoles were significantly enlarged in *Δpqr1* 24 h after −N, compared with WT (*p* < 0.01, N = 52). In *Δpqr1*, nuclei were not located centrally, but peripherally, probably due to vacuolar enlargement in *Δpqr1* (Fig. 1*C*). Then vacuolar functions in *Δpqr1* were examined using GFP-tagged Cpy1 carboxypeptidase Y, which requires functional vacuolar proteases for its maturation (Fig. S2) (33, 34). The level of immature Cpy1-GFP was higher in *Δpqr1* than in WT, suggesting that maturation of Cpy1 may be impaired in *Δpqr1* to some extent.

Interestingly, the severe lethality of *pqr1*^*+*^ deletion under −N was almost completely suppressed by simultaneous deletion of *atg* genes, (*atg3*^*+*^, *atg5*^*+*^, *atg6*^*+*^, *atg7*^*+*^, *atg8*^*+*^, or *atg13*^*+*^), all of which are indispensable for autophagosome formation (Fig. 2*F*). While the viability of *Δpqr1* 72 h after −N was ∼4%, all double mutants *Δpqr1Δatg* maintained high viability (>70%) comparable to that of WT (∼84%). Single-deletion mutants of *atg* genes did not show significant viability loss within 72 h after −N, consistent with previous studies (8, 9, 35). In a previous study (35), a similar loss of viability of *Δisp6* after −N was suppressed by *Δatg*. Isp6 is an ortholog of the *Saccharomyces cerevisiae* vacuolar proteinase B Prb1 and is required for autophagy-dependent protein degradation induced by −N (36).

### TEM observation of vacuolar morphology

**Figure 3:**
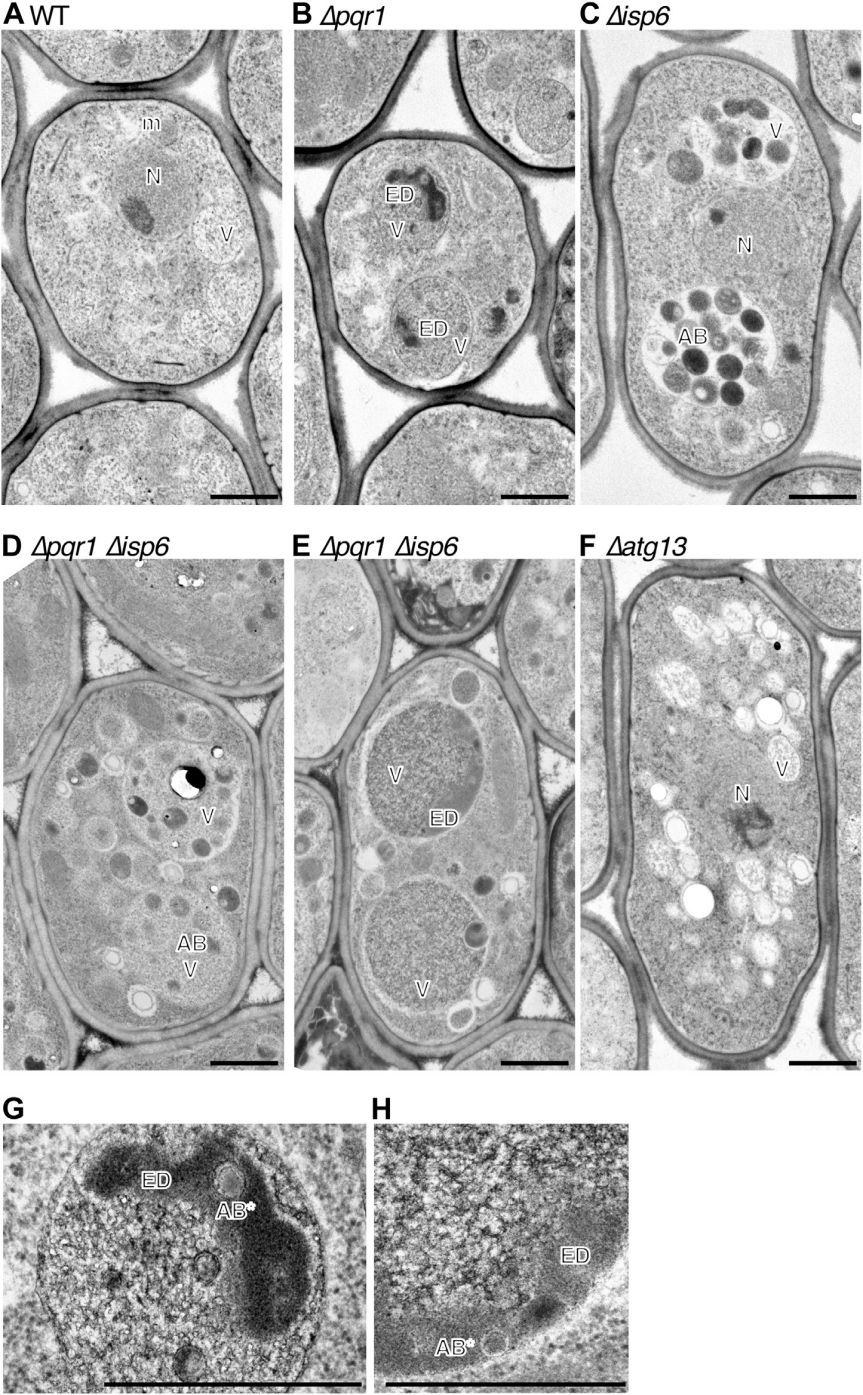
**Electron microscopic analyses on morphology of vacuole under −N condition.** WT (*A*), *Δpqr1* (*B* and *G*), *Δisp6* (*C*), *Δpqr1Δisp6* (*D*, *E* and *H*), and *Δatg13* (*F*) cells were harvested 7 h after −N and observed by TEM. *G* or *H* is a magnified image of *B* or *E*, respectively. Indications for cellular structures are as follows; AB, autophagic body; AB∗, autophagic body-like structure; ED, electron-dense amorphous materials (EDAM); m, mitochondrion; N, nucleus; V, vacuole. Bar, 1 μm.

To obtain further information on vacuoles, we performed electron microscopic analyses, adopting freeze-substitution methods (Fig. 3). Cells of WT, *Δpqr1*, *Δisp6*, *Δpqr1Δisp6*, and *Δatg13* were harvested 7 h after −N and were observed. While well-developed vacuoles were seen in WT, vacuoles of *Δisp6* contained a number of autophagic bodies (AB), consistent with a previous report (35). Interestingly, in vacuoles of *Δpqr1*, ABs were not obviously accumulated comparing with *Δisp6*, but instead, unidentified electron-dense, amorphous materials (hereafter EDAM) without membranes were seen (Fig. 3, *B* and *H*, indicated by ED). Other than EDAMs, the vacuolar lumen of *Δpqr1* was much more electron-densely stained than in WT. In *Δpqr1Δisp6*, ABs were accumulated and EDAMs were also observed (Fig. 3, D, E and I). The AB accumulation in *Δpqr1Δisp6* suggests that either autophagosome formation or autophagosome-vacuole fusion may occur in the absence of *pqr1*^*+*^.

### Pqr1 shares protein structure with *Arabidopsis* NLA, important for regulation of phosphate uptake

**Figure 4:**
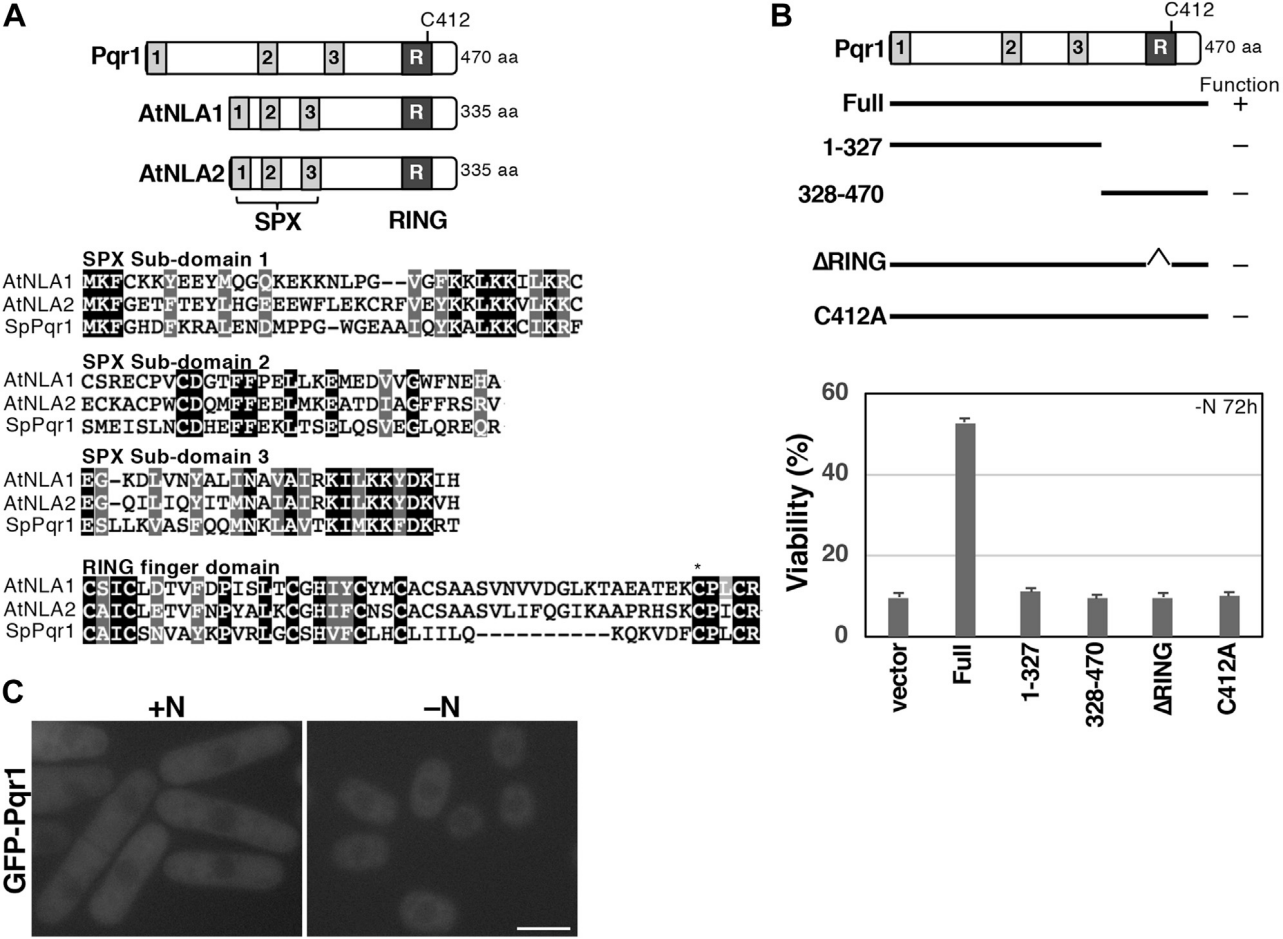
**Pqr1 is similar to the ubiquitin ligase, NLA, of *A. thaliana*, having an SPX domain and a RING finger.***A*, schematic drawing of *S. pombe* Pqr1 and *A. thaliana* NLA and NLA2. *Gray boxes* 1 to 3 indicate subdomains of the SPX domain. *Darker boxes* indicate RING finger motifs (*top*). Alignments of NLA, NLA2 and Pqr1. *B*, domain analyses of Pqr1. Both the SPX and the RING finger were indispensable for the function of Pqr1. Plasmids expressing the indicated region of Pqr1 were introduced into *Δpqr1* and viability was measured 72 h after −N. Experiments were repeated 3×, and means and SDs are presented. *C*, localization of GFP-Pqr1 in ±N. Bar, 5 μm.

Pqr1 possesses a C_3_HC_4_-type RING (Really Interesting New Gene) finger motif in the C-terminal region and an SPX (Syg1-Pho81-Xpr1) domain in the N-terminal region (Fig. 4*A*). It has been suggested that the SPX domain may act as a “phosphate sensor” by binding inositol polyphosphates (InsPs), concentrations of which may respond to changes in cellular phosphate availability (27, 28, 29, 30, 31). Therefore, the SPX domain is found in proteins involved in phosphate regulation and metabolism. The structure of Pqr1 is closely related to that of a ubiquitin ligase NLA (Nitrogen Limitation Adaptation) of *Arabidopsis thaliana*, genetically identified as a protein required for normal growth under limited nitrogen (37). *nla* mutants show no abnormalities in nitrogen-rich soil, implying a similarity between *nla* mutants and *S. pombe Δpqr1*, in which viability is lost only under −N. Later studies reported that NLA is engaged in restriction of phosphate uptake *via* ubiquitination, subsequent endocytosis, and intravacuolar degradation of the high-affinity phosphate transporter PHT1 (38).

We performed domain analysis of Pqr1 (Fig. 4*B*). Truncated proteins (1–327: SPX only, 328–470: SPX deleted, ΔRING: RING finger deleted) and an amino acid substitution mutant (C412A: essential cysteine C412 in the RING finger was replaced by alanine) were created and cloned into the plasmid expression vector, Rep41, under control of the inducible nmt41 promoter, activated by thiamine withdrawal. We introduced an expression vector harboring mutant Pqr1 into *Δpqr1* and examined whether viability of *Δpqr1* in −N is restored. No mutant proteins rescued *Δpqr1* lethality in −N, suggesting that both SPX and RING are required for proper function of Pqr1.

### GFP-Pqr1 is localized to the cytoplasm

To examine localization of Pqr1, we created a strain expressing GFP-Pqr1 controlled by the nmt41 promoter. The expression cassette was integrated into the *leu1*^*+*^ locus of chromosome II. Expression of GFP-Pqr1 was induced in ±N and examined by fluorescent microscopy (Fig. 4*C*). GFP-Pqr1 was localized throughout the cytoplasm under ±N conditions.

### Reducing the phosphate level in the medium suppresses the lethality of *Δpqr1* in −N

Given that Pqr1 is structurally similar to NLA, a regulator of cellular phosphate uptake, phenotypes of *Δpqr1* could be affected by changing the phosphate concentration in the medium. To explore this possibility, a new medium, EMM2–N without phosphate, was introduced. The original EMM2 contains 15.5 mM phosphate as Na_2_HPO_4_, which is for adjusting the pH of the medium to ∼pH 5.8. When Na_2_HPO_4_ is completely removed, the pH of the new medium falls below 4.0, which could affect cellular homeostasis. To avoid this, 10 mM 2-(*N*-morpholino) ethanesulfonic acid (MES) was added to EMM2–N without phosphate and the pH was adjusted with NaOH. We designate this new medium as EMM2–NP.

**Figure 5:**
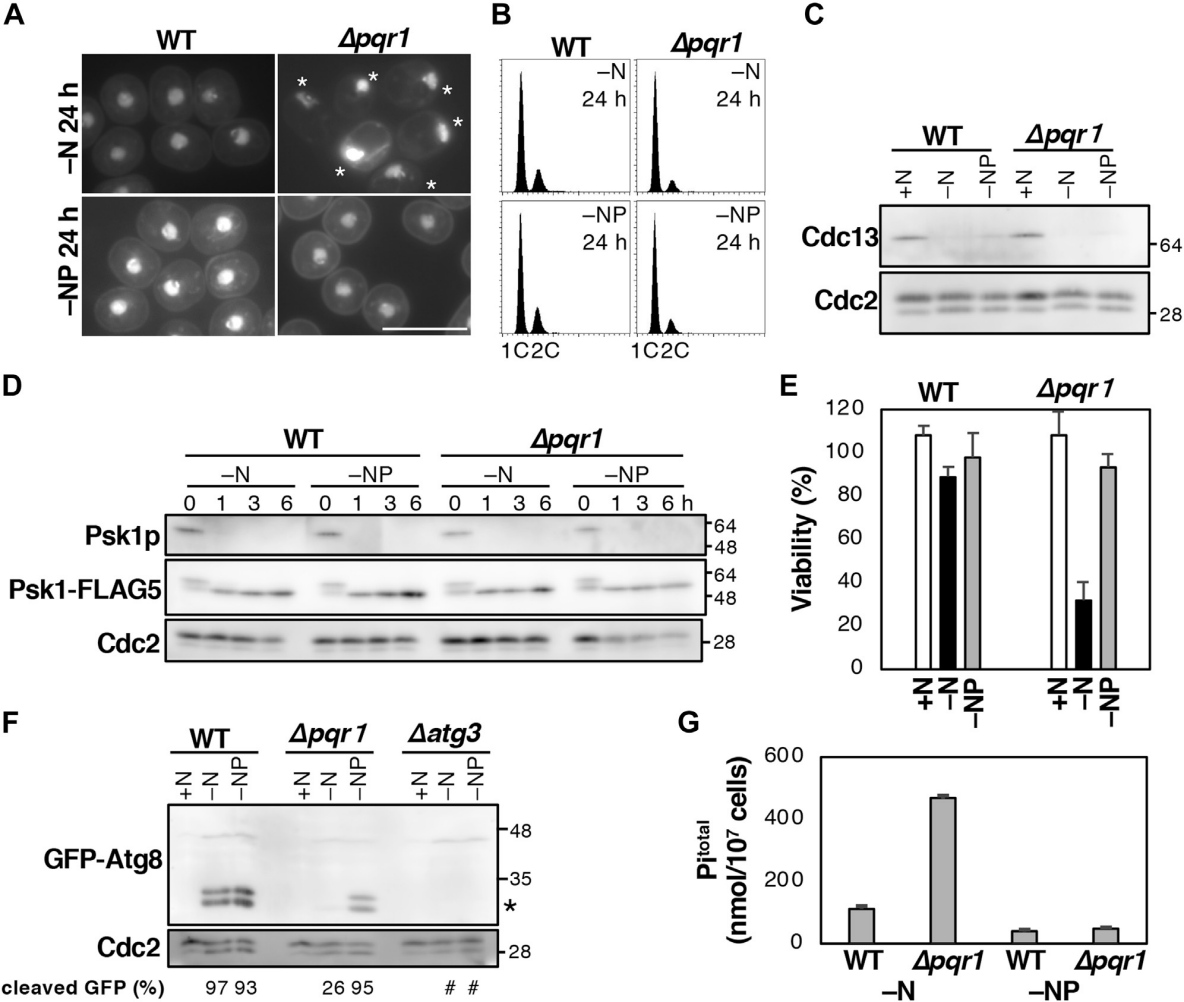
**Abnormal phenotypes of *Δpqr1* were suppressed by reducing the phosphate concentration of the medium.***A*, cellular morphologies of indicated strains at 24 h after −N and −NP were observed with DAPI staining. Note the abnormal nuclear localization of *Δpqr1* (asterisks) under −N, but not −NP conditions. Bar, 10 μm. *B*, both WT and *Δpqr1* arrested the cell cycle with 1C DNA content 24 h after −N and −NP. *C*, degradation of the mitotic cyclin, Cdc13. *D*, time-course analysis of dephosphorylation of the TORC1 target, Psk1, in WT and *Δpqr1* after −N and −NP. *E*, viability of WT and *Δpqr1* under +N, −N, and −NP. *F*, autophagy assays using GFP-Atg8. Cleaved GFP (*asterisk*) was detected clearly in *Δpqr1* under −NP. Cleaved GFP bands in *Δatg13* were difficult to be quantified due to backgrounds (sharp). *G*, total amounts of intracellular phosphate in WT and *Δpqr1* were measured 24 h after −N and −NP. For *E* and *G*, experiments were repeated 3×, and means and SDs are presented.

WT cells were cultured to log phase in EMM2 at 26 °C and shifted to EMM2–N or EMM2–NP. Then, cellular concentration, cell shape, ploidy, and viability were determined (Fig. 5 and Fig. S1). No obvious differences were observed between EMM2–N and EMM2–NP. WT cells divided approximately twice, arrested with 1C DNA content, and reduced cell size at 24 h after either −N or −NP. Reduction of Cdc13 level and inactivation of TORC1 (dephosphorylation of Psk1) occurred in EMM2–NP as well (Fig. 5, *C* and *D*). *Δpqr1* mutants were examined similarly in EMM2–N and EMM2–NP. No differences were seen in *Δpqr1*, except for viability in quiescence and the location of nuclei. *Δpqr1* mutants in EMM2 or cultured for 24 h in EMM2–N or EMM2–NP displayed viability of ∼100%, 30%, and 93% respectively (Fig. 5*E*). Similarly, the abnormal nuclear location seen in *Δpqr1* in −N was also completely suppressed in −NP (Fig. 5*A*). Next, autophagy-dependent proteolysis in EMM2–NP was analyzed using the GFP-Atg8 method. Cleaved GFP was detected in WT in −N and −NP, while it was hardly detected in *Δpqr1* in −N, as previously described. In contrast, cleaved GFP was clearly detected in *Δpqr1* in −NP (Fig. 5*F*). Phenotypes of *Δpqr1* in −N were almost completely suppressed by removing phosphate from the medium, much as abnormalities of NLA-defective *Arabidopsis* mutants, apparent in N-poor conditions, were suppressed by reducing phosphate (39). *S. pombe* Pqr1 is likely a structural and functional ortholog of *Arabidopsis* NLA, the regulator of phosphate uptake.

### *Δpqr1* cells contain more phosphate than WT cells

We then measured the total amount of intracellular phosphate (Pi^total^) (Fig. 5*G*). WT and *Δpqr1* cells were cultured in EMM2 and shifted to EMM2–N or EMM–NP for 24 h. Harvested cells were treated with 1 M H_2_SO_4_ at 95 °C for 30 min to hydrolyze all phosphate residues in the cells, and phosphate released into the supernatant was quantified by the malachite green method (40). The Pi^total^ per 10^7^ cells was calculated (Fig. 5*G*). While Pi^total^ of WT and *Δpqr1* in EMM2–N were 112 and 466 nmol/10^7^ cells, respectively, quantities in EMM2-NP were 38 (WT) and 49 (*Δpqr1*). To compare phosphate uptake amount, we performed time-course analysis of Pi^total^ of WT and *Δpqr1* after −N (Fig. S3). Because cell size is reduced after −N (Fig. 1*C*), Pi^total^ values were normalized by wet cell weight (mg). Although Pi^total^ was increased in both WT and *Δpqr1* after −N, Pi^total^ increase in *Δpqr*1 was significantly greater than in WT (*p* < 0.001), suggesting that *Δpqr1* may incorporate phosphate more than WT. Pi^total^ levels and suppression of *Δpqr1* phenotypes by reduction of phosphate levels suggest that hyperaccumulation of phosphate may cause a severe viability loss and defects in autophagy-dependent proteolysis in *Δpqr1*.

### High-affinity phosphate transporters interact genetically with *pqr1*^+^

Inorganic phosphate in the medium is incorporated into cells *via* phosphate transporters in the plasma membrane (41). *S. pombe* possesses four genes encoding high-affinity phosphate transporters (*pho84*^*+*^ (42), SPBC1683.01, SPAC23D3.12, and SPCC2H8.02) and one encoding a low-affinity phosphate transporter (SPBC3B8.04c). Hereafter we designate SPBC1683.01, SPAC23D3.12, SPCC2H8.02, and SPBC3B8.04c as *pho841*^*+*^, *pho842*^*+*^, *pho843*^*+*^, and *plt1*^*+*^ (*plt*: phosphate low-affinity transporter), respectively (Fig. S4*A*).

**Figure 6:**
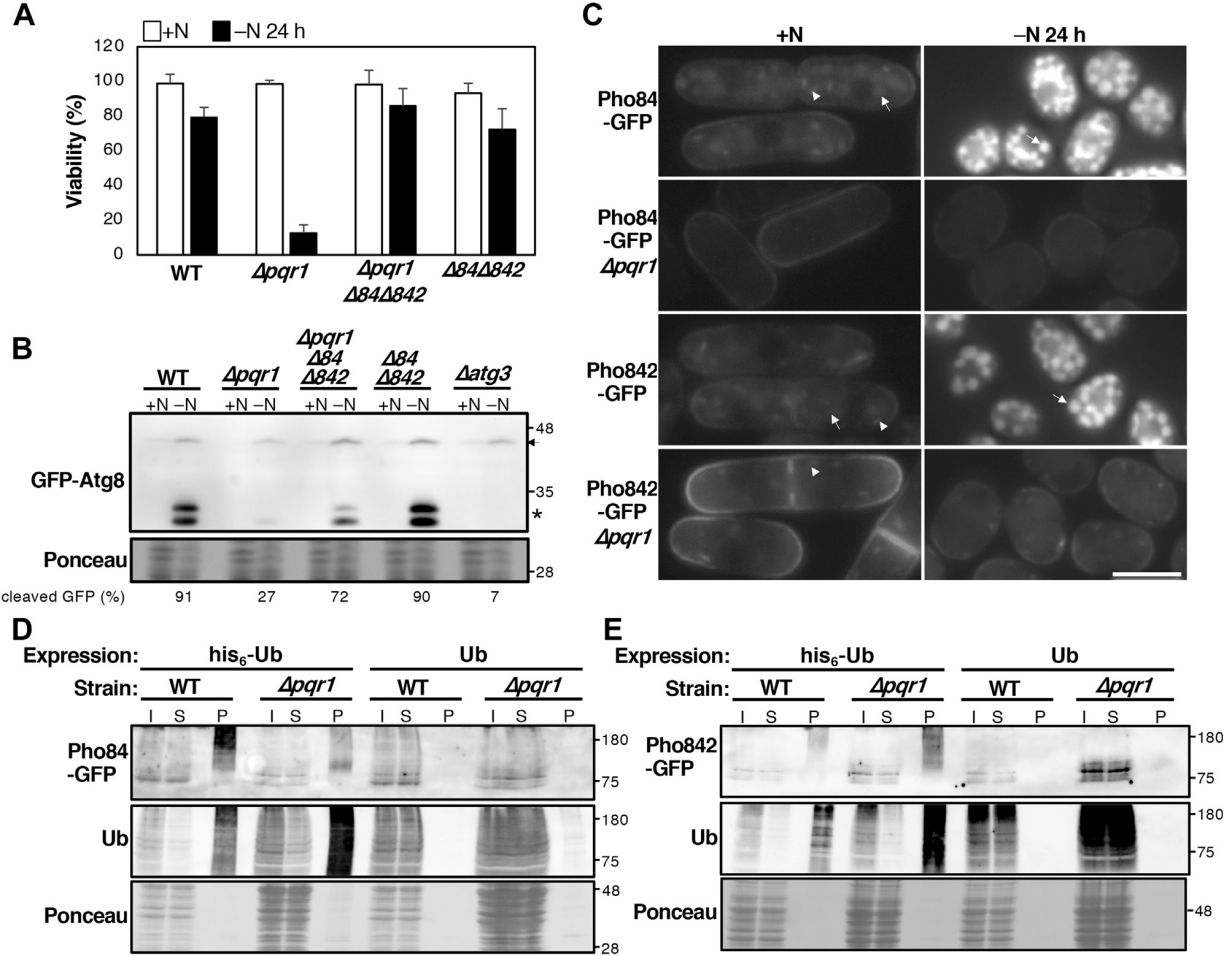
**Pqr1 is required for proper localization of high-affinity phosphate transporters, Pho84 and Pho842.***A*, viabilities of indicated strains at 0 and 24 h after −N. The viability loss of *Δpqr1* was suppressed by double gene deletion, *Δpho84Δpho842*. *Δ84* and *Δ842* indicate *Δpho84* and *Δpho842*, respectively. Experiments were repeated 3×, and means and SDs are presented. *B*, autophagy assays using GFP-Atg8. Cleaved GFP (*asterisk*) was detected in *Δpqr1Δpho84Δpho842* 24 h after −N, but not in *Δpqr1*. The *arrow* indicates GFP-Atg8. *C*, intracellular localization of Pho84-GFP and Pho842-GFP was affected by *Δpqr1*. Under +N, Pho84 and Pho842 were localized to the cell periphery and vacuoles in WT, while they were mainly localized in vacuoles 24 h after −N. In *Δpqr1*, both Pho84 and Pho842 were localized mainly to the cell periphery under ±N conditions. See Fig. S5 for colocalization of Pho84/842 and vacuoles. *Arrowheads*, unidentified structures. Bar, 5 μm. *D*, analysis of Pho84 ubiquitination. his6-Ub and Ub were overproduced from plasmids in WT and *Δpqr1* expressing Pho84-GFP. Ubiquitinated proteins were purified using Ni-beads from cell extracts prepared under denaturing conditions with 8 M urea. Pho84-GFP and ubiquitinated proteins were detected by immunoblot. I, S, and P indicate input, supernatant, and precipitated proteins, respectively. 5.5× more sample was loaded for P than for I and S. *E*, analysis of Pho842 ubiquitination.

Each *S. pombe* phosphate transporter gene was individually deleted using KANMX marker genes and all deletion mutants were viable, consistent with the comprehensive gene deletion study (43). If Pqr1 is involved in restricting phosphate uptake by downregulating phosphate transporters, as NLA is, we suspected that deleting phosphate transporter genes might affect the phenotypes of *Δpqr1*. We combined *Δpqr1* with single-gene deletions of phosphate transporters, *Δpho84*, *Δpho841*, *Δpho842*, *Δpho843*, or *Δplt1*, and found that the viability loss phenotype of *Δpqr1* was not affected (Fig. S4*B*). Then *Δpqr1* was combined with double deletions of phosphate transporters and viabilities of ten triple mutants were determined. For the first trial, five triple mutants among the ten maintained viability >40%, 24 h after −N, while the viability of *Δpqr1* was ∼15% (Fig. S4*C*). Then, the same experiment was repeated 3×, and we found that only *Δpqr1Δpho84Δpho842* reproducibly sustained high viability comparable to WT (Fig. 6*A* and Fig. S4*D*). Next, we examined autophagy-dependent proteolysis using the GFP-Atg8 method (Fig. 6*B*). While cleaved GFP was faintly detected in *Δpqr1* in −N, it was strongly detected in *Δpqr1Δpho84Δpho842*. These results suggest that gene deletion of *pho84*^*+*^ and *pho842*^*+*^ may restrict phosphate uptake from the medium, rescuing the phenotype of *Δpqr1* caused by excess phosphate uptake.

### Pho84 and Pho842 change localization after nitrogen source withdrawal

To explore intracellular localization of Pho84 and Pho842, the GFP gene was C-terminally fused to endogenous *pho84*^*+*^ or *pho842*^*+*^. In WT cells cultured in +N, both Pho84 and Pho842 were localized peripherally, probably to the plasma membrane and to vacuoles, and also to unidentified structures (Fig. 6*C* and Figs. S5 and S6). Interestingly, in WT, the two phosphate transporters translocated to vacuoles 24 h after −N. As the signals of Pho84-GFP and Pho842-GFP in WT 24 h after −N were too strong to compare with those in +N, images were digitally adjusted (Fig. 6*C*). Images in the left column (+N) were digitally enhanced in the same way, while those in the right column (−N) were not modified (for details, see Fig. S5). The drastic changes in localization of Pho84 and Pho842 depended on Pqr1 function. In *Δpqr1* cells, both remained localized to the plasma membrane, even 24 h after −N (Fig. 6*C*). To avoid the halation caused by hyperaccumulated GFP in vacuoles, we utilized a Super-Ecliptic pHluorin (SEpH), which loses its fluorescence in low-pH environments such as vacuolar lumens (44), to visualize Pho84 and Pho842 (Fig. S7), and we obtained data consistent with result described above. Reduction of SEpH fluorescence in vacuoles in WT and *Δpqr1* also suggested that vacuolar acidity, important for protease functions (45), may be normal in *Δpqr1*. These data are consistent with the result that phosphate uptake is much greater in *Δpqr1* than WT (Fig. S3). Hyperaccumulation of phosphate in *Δpqr1* is likely due to retention of Pho84 and Pho842 on plasma membrane. Considering the foregoing results and previous reports, it appeared that Pqr1-mediated downregulation of phosphate transporters, which may be important to sustain viability and autophagy-dependent proteolysis in −N, by restricting phosphate uptake.

### Ubiquitination of Pho84 depends mainly on Pqr1

As Pqr1 is a probable ubiquitin ligase, we examined whether Pqr1 ubiquitinates high-affinity phosphate transporters. First, either hexa-histidine-tagged ubiquitin (his6-Ub) or ubiquitin (Ub) was overexpressed from multicopy plasmids with the strong inducible promoter, nmt1, in WT and *Δpqr1* cells expressing Pho84-GFP. After inducing expression of his6-Ub or Ub for 20 h at 26 °C, cells were harvested and proteins were extracted in denaturing lysis buffer containing 8 M urea to destroy noncovalent protein interactions. Then extracts were incubated with Ni-agarose beads to purify his6-ubiquitinated substrates and subsequently analyzed by immunoblot to determine whether Pho84-GFP was ubiquitinated (Fig. 6*D*). In WT cells expressing his6-Ub, Pho84-GFP was detected, showing a smeared electrophoretic pattern in the Ni-purified fraction (P). In *Δpqr1* expressing his6-Ub, Pho84-GFP was detected, but it was greatly reduced in the Ni-purified fraction. In either WT or *Δpqr1*, Pho84-GFP signals were not detected by Ub expression in the Ni-purified fraction (P) of negative controls. These results imply that: (1) Pho84 is multiply ubiquitinated; (2) ubiquitination of Pho84 depends mainly on Pqr1.

The same ubiquitination assay was performed with Pho842 (Fig. 6*E*). Pho842-GFP was detected as a smear in the his6-Ub purified fraction in WT, suggesting that Pho842-GFP was also multiubiquitinated. Unexpectedly, we detected Pho842-GFP in the his6-Ub purified fraction of *Δpqr1*, suggesting that the major ubiquitin ligase for Pho842 is not Pqr1, although we cannot exclude the possibility that Pqr1 participates in its ubiquitination as well.

### The VTC complex is required for polyP synthesis in *S. pombe*

In fungi, phosphate is stored in vacuolar lumens as polyphosphate (polyP). In *S. cerevisiae*, the VTC (vacuolar transporter chaperone) complex on vacuolar membranes synthesizes polyP into the vacuolar lumen using cytoplasmic ATP as a substrate (46). There are two types of VTC complex in *S. cerevisiae*. One consists of Vtc1, Vtc2, and Vtc4 and another consists of Vtc1, Vtc3, and Vtc4. In both, Vtc4 serves as the catalytic subunit. Vtc2, Vtc3, and Vtc4 possess an SPX domain at the N-terminus, and activity of the VTC complex is affected by cytoplasmic phosphate concentration (47). *S. pombe* possesses orthologs of VTC subunits, Vtc2 and Vtc4. *S. cerevisiae* Vtc1 is structurally similar to *S. pombe* Nrf1 (48).

**Figure 7:**
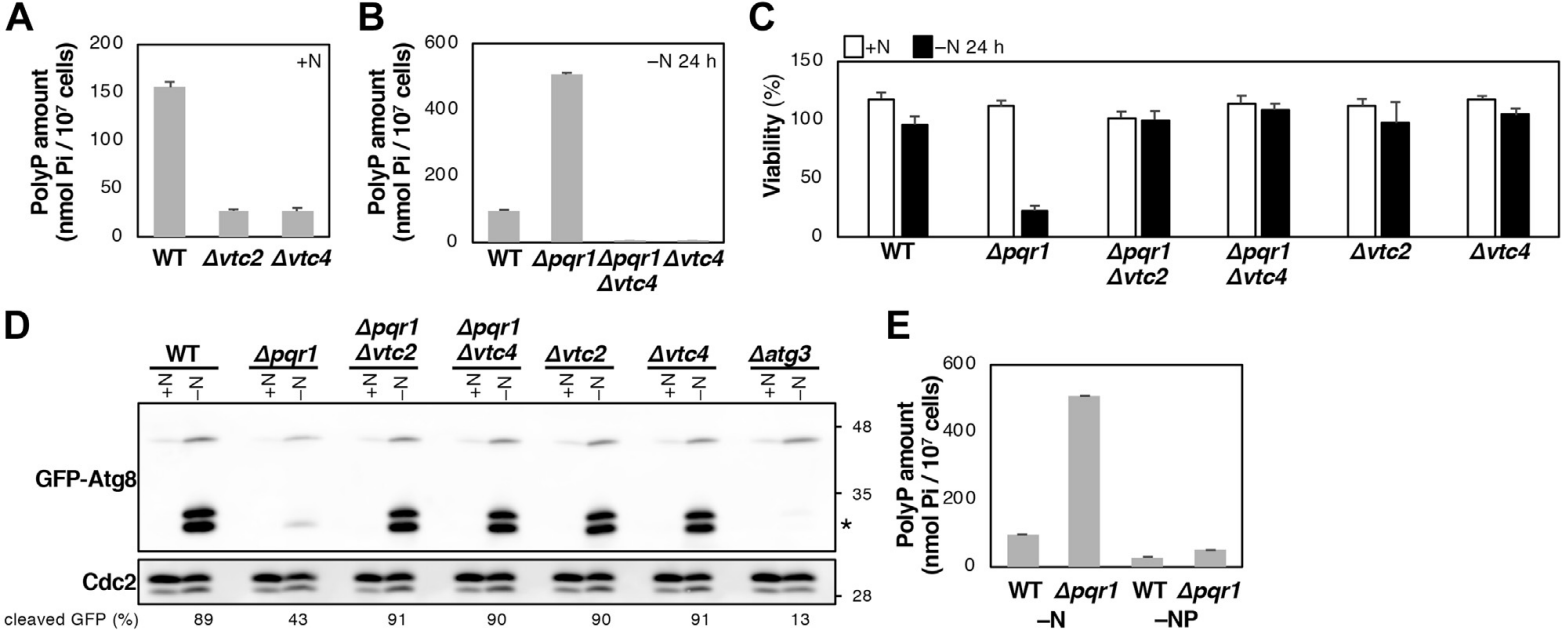
**Hyper-accumulation of polyP causes defective autophagy-dependent proteolysis.***A*, Vtc2 and Vtc4 are responsible for intracellular polyP in *S. pombe*. *B*, intracellular polyP amounts were measured in indicated strains 24 h after −N. *C*, viabilities of indicated strains. *Δpqr1* lost viability under −N conditions, but *Δpqr1Δvtc2* and *Δpqr1Δvtc4* did not. *D*, autophagy assay using GFP-Atg8. Cleaved GFP (*asterisk*) was scarce in *Δpqr1* under −N, although clearly detected in *Δpqr1Δvtc2* and *Δpqr1Δvtc4*. *E*, intracellular polyP was measured in WT and *Δpqr1* 24 h after −N or −NP. Analyses of *B* and *E* were performed at the same time. For *A*–*C* and *E*, experiments were repeated 3×, and means and SDs are presented.

Because phenotypes of *Δpqr1* could be caused by excess phosphate uptake, we explored the relationship between *Δpqr1* phenotypes and polyP stored in vacuolar lumens, where autophagic proteolysis occurs. First, *vtc2*^*+*^ and *vtc4*^*+*^ genes were deleted using a KANMX drug-resistant marker gene and *Δvtc2* and *Δvtc4* proved viable, consistent with a comprehensive gene deletion study (43). We then quantified intracellular polyP in WT, *Δvtc2*, and *Δvtc4* cells cultured in EMM2+N. Purified polyP was hydrolyzed by 1 M H_2_SO_4_ treatment at 95 °C for 30 min and released phosphate was quantified using the malachite green method. Then we calculated how much phosphate existed as polyP in 10^7^ cells (Fig. 7*A*). While 10^7^ WT cells contained 155 ± 6 nmol of phosphate as polyP, *Δvtc2* cells contained 27.1 ± 2 nmol, and *Δvtc4* cells contained 26.8 ± 3 nmol (N = 3: means and SD). This clearly indicates for the first time that the *S. pombe* VTC complex synthesizes polyP, as in *S. cerevisiae*.

### Reduced polyP suppressed the viability loss and autophagic proteolysis defect in *Δpqr1*

Intracellular polyP in WT, *Δpqr1*, *Δpqr1Δvtc4*, and *Δvtc4* cultured in EMM2–N for 24 h was quantified (Fig. 7*B*). PolyP accumulated fivefold more in *Δpqr1* than in WT, and the hyperaccumulation of polyP in *Δpqr1* was completely suppressed by simultaneous deletion of *vtc4*^*+*^ (Fig. 7*B*). Comparison of Pi^total^ and polyP was performed using these strains, and we found that the majority of Pi^total^ is polyP (Fig. S8). Amounts of cellular phosphate other than polyP were not significantly different among WT, *Δpqr1*, *Δpqr1Δvtc4*, and *Δvtc4* (Fig. S8). Then we examined viabilities of these strains. Twenty-four hours after −N, the severe viability loss in *Δpqr1* was almost completely suppressed by either *vtc2*^*+*^ or *vtc4*^*+*^ deletion (Fig. 7*C*). Hence, we conclude that inactivation of the VTC complex suppresses the *Δpqr1* viability loss.

Next, using the GFP-Atg8 method, we examined whether autophagy-dependent proteolysis is restored in *Δpqr1* cells by VTC complex inactivation. While cleaved GFP was faintly detected in *Δpqr1* in −N, levels of cleaved GFP in *Δpqr1Δvtc2* and *Δpqr1Δvtc4* were comparable to that in WT (Fig. 7*D*), indicating that inactivating the VTC complex restored autophagy-dependent proteolysis compromised in *Δpqr1*. As levels of cleaved-GFP in *Δvtc2* and *Δvtc4* cells were the same as in WT, the VTC complex may not be required for starvation-induced autophagy in *S. pombe*.

### PolyP level was reduced in EMM2–NP

Lethality and the defect in autophagy-dependent proteolysis in *Δpqr1* were suppressed in EMM2–NP (Fig. 4). We examined whether intracellular polyP level is reduced in EMM2–NP (Fig. 7*E*). WT and *Δpqr1* cells were cultured in EMM2 and shifted to EMM2–N or EMM2–NP for 24 h to purify and quantify polyP. WT and *Δpqr1* cells in EMM2–N contained 95.6 ± 3 and 507 ± 5 nmol of phosphate as polyP in 10^7^ cells, while WT and *Δpqr1* cells in EMM2–NP possessed 28.8 ± 4 and 49.0 ± 3 nmol phosphate/10^7^ cells. Hyperaccumulation of polyP in *Δpqr1* was suppressed in EMM2–NP, even if subunits of the VTC complex were not mutated.

## Discussion

**Figure 8:**
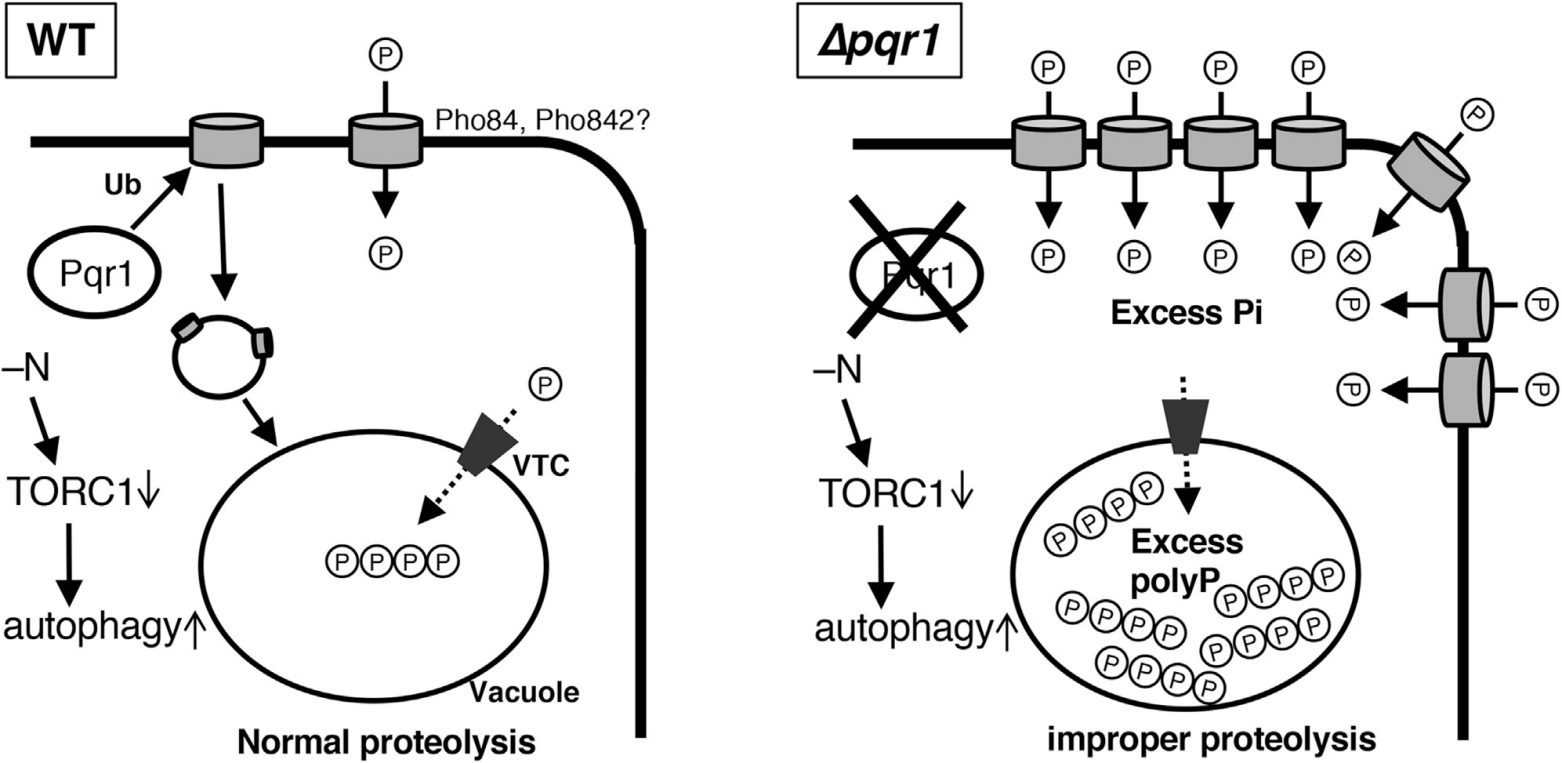
**Working hypothesis.** In WT, Pho84 and Pho842 on the plasma membrane are decreased by Pqr1 function upon −N to restrict phosphate uptake. The vacuole contains polyP at a normal level and is able to degrade autophagosomes. Without Pqr1, Pho84 and Pho842 remain on plasma membrane and incorporate too much phosphate, resulting in hyperaccumulation of polyP, which interferes with proper autophagy-dependent proteolysis.

In the present study, we examined molecular functions of an SPX-RING ubiquitin ligase (Pqr1) in *S. pombe*. The major outcomes were as follows: Pqr1 is essential for cellular viability under −N-induced quiescence by maintaining proper vacuolar environments and guaranteeing autophagy-dependent proteolysis. Pqr1 is required for internalization of the phosphate transporters, Pho84 and Pho842, from plasma membranes. Inactivation of Pqr1 induces hyperaccumulation of intracellular polyP and total phosphate. Furthermore, abnormalities in *Δpqr1*, defective autophagy-dependent proteolysis and viability loss, are suppressed by reduction of polyP. Our results suggest that appropriate levels of intracellular polyP are essential for vacuolar functions required for autophagy-dependent proteolysis and for survival during quiescence (Fig. 8). Upon −N, phosphate transporters Pho84 and Pho842 are downregulated by Pqr1 and phosphate uptake is restricted, so that intracellular polyP is controlled to maintain normal vacuolar functions. If Pqr1 is deficient, hyperuptake of phosphate may occur, since Pho84 and Pho842 remain on the plasma membrane after −N (Fig. S3 and Fig. 6). Due to excess phosphate influx into the cytoplasm, synthesis of polyP may be accelerated to prevent high free phosphate concentrations, resulting in hyperaccumulation of polyP, which apparently interferes with vacuolar functions including proteolysis, although the precise molecular mechanism remains unknown.

Our study suggests that Pqr1 is a functional ortholog of *Arabidopsis* NLA, an SPX-RING ubiquitin ligase, originally identified as the gene responsible for *nla* mutants, which shows growth abnormalities only on nitrogen-poor soils (37) and is a key regulator of phosphate uptake *via* ubiquitination, endocytosis, and subsequent vacuolar degradation of PHT1, a high-affinity phosphate transporter (38). Phenotypes of *nla* mutants on nitrogen-poor soils are suppressed by lowering phosphate concentration in the soil and also by mutations of PHT1 (49). It was also reported that the 26S proteasome may be involved in degradation of PHT1 (50). Our data strongly suggest that Pqr1 regulates trafficking of phosphate transporters *via* ubiquitination as well. As another possibility is that Pqr1 could be involved in efficiency of vacuolar proteolysis of the transporters; hence, a Pqr1 defect could affect their localization. In this study, we could not address whether Pqr1 regulates other phosphate transporters, Pho841, Pho843, and Plt1, because expression levels of these three were too low (Fig. S4 and unpublished results). SPX-RING proteins are found in other fungi, except for budding yeast, but not in metazoa. In *S. cerevisiae*, endocytosis of Pho84 (high-affinity phosphate transporter), Pho87, and Pho90 (low-affinity phosphate transporters) depends on ubiquitination (51, 52). The ubiquitin ligase for Pho87 and Pho90 is thought to be Rsp5, while that for Pho84 has not been identified.

Ubiquitination of Pho84 and Pho842 was examined in this study. While ubiquitination of Pho84 was largely dependent on Pqr1, Pho842 apparently was ubiquitinated in *Δpqr1*. A previous study demonstrated that five lysine residues in Pho84 (K13, K265, K273, K288, and K321) and four in Pho842 (K9, K13, K40, and K260) are ubiquitinated (53). K265, K273, K288, and K321 of Pho84 and K260 of Pho842 are located in a long cytoplasmic loop between the sixth and seventh transmembrane regions (central loop, Fig. S4). It was reported that ubiquitination of lysine residues in the cytoplasmic loop of *S. cerevisiae* Pho84 may be required for its endocytosis-dependent internalization (52). Accordingly, Pho842 could be ubiquitinated by Pqr1 on K260 in the central loop and the other three lysines (K9, K13, and K40) could be targets of other ubiquitin ligases. *S. pombe* amino acid transporters are ubiquitinated by a HECT-type ubiquitin ligase, Pub1, an ortholog of Rsp5 in budding yeast, and they are internalized, depending on Arrestin-related adaptors (54, 55, 56). Experiments using mutants of Pho84 and Pho842, in which lysine residues are substituted with arginine, are of great interest to evaluate physiological effects of ubiquitination.

In *S. pombe*, transcription of the *pho84*^*+*^ gene is activated and Pho84 is more enriched on plasma membranes during phosphate starvation (42) (Takeda, unpublished). In this study, Pho84 and Pho842 drastically alter their localization to vacuoles in a Pqr1-dependent manner after a shift to −N, although phosphate level in the medium was identical. Downregulation of Pho84 and Pho842 by Pqr1 upon −N might contribute to balancing between cellular nitrogen and phosphate through restricting phosphate uptake. Molecular mechanisms by which changes in nitrogen level affect behaviors of phosphate transporters were not further examined in the present study. Under −N, TORC1 is inactivated immediately, affecting many metabolic processes, including vesicular trafficking (57, 58). In *S. cerevisiae*, administration of the TORC1 inhibitor, rapamycin, internalizes Pho87, a low-affinity phosphate transporter (51). Hence, it will be of interest to explore whether TORC1 participates in localization of phosphate transporters and in the activity of *S. pombe* Pqr1.

Pqr1 substrates other than phosphate transporters were not investigated in the present study; however, this possibility should be considered in the future. In *S. cerevisiae*, the SPX protein, Pho81, participates in the response to phosphate starvation (the PHO pathway) by inhibiting activity of Pho85/Pho80, the CDK/cyclin complex that phosphorylates and regulates the transcription factor Pho4 (59, 60, 61). Pho4 induces expression of genes that increase phosphate uptake during phosphate starvation (secreted phosphatases and Pho84). Although the PHO pathway has been intensively studied, the central regulator, Pho81, is not widely conserved in other eukaryotes, including *S. pombe* (62, 63, 64). *S. pombe* possesses six SPX proteins. Of these, Pqr1 and Gde1 (glycerophosphoryl diester phosphodiesterase) are cytoplasmic proteins, such as Pho81 in *S. cerevisiae*, while the other four are transmembrane proteins. Given that the SPX domain may serve as a phosphate sensor, Pqr1, a cytoplasmic ubiquitin ligase may have substrates other than phosphate transporters and may coordinate cellular responses to changes in phosphate availability. Investigation of Pqr1 substrates could further illuminate roles of NLA in phosphate regulation in plants.

Our results strongly suggest that the lethality of *pqr1*^*+*^ deletion may be due to defects in autophagy-dependent degradation in vacuoles. Although it seems counterintuitive, deletion mutants of *atg* genes required to trigger autophagy maintain high viability (∼70%) for several days after −N. This viability decreases gradually, in sharp contrast to *Δpqr1*, which loses viability abruptly (within 24 h) (8, 9, 35). Furthermore, the present study showed that the lethality of *Δpqr1* was suppressed by deletion of *atg* genes. These results indicate that the lethality of *pqr1*^*+*^ deletion depends on autophagosome formation.

Deletion of *isp6*^+^ has been reported to phenocopy *Δpqr1*. Isp6 in *S. pombe* is the counterpart of *S. cerevisiae* Prb1, a serine protease in vacuoles required to activate other vacuolar proteases and therefore essential for autophagy-dependent proteolysis under nitrogen starvation (65). *Δisp6* cells lose viability due to the insufficiency of vacuolar protease activity after −N, and lethality is suppressed by simultaneous deletion of *atg* genes, as seen in *Δpqr1* (35). In order to explain the genetic interaction between *Δisp6* and *Δatg*, two possibilities were discussed by Kohda *et al.* (2007). (1) Accumulation of ABs in vacuoles, which depends on an active autophagy pathway, may have toxic effects in *Δisp6*; (2) *Δisp6* cells may accelerate autophagy due to a deficiency of autophagy-dependent amino acid recycling under −N, such that essential cytoplasmic components may be encompassed by ABs (35).

Despite the phenotypic similarity of *Δisp6* and *Δpqr1*, TEM analysis revealed a difference in accumulation of AB and EDAM, an unidentified structure shown in Figure 3. In *Δpqr1*, autophagosomes may be formed normally and incorporated into vacuoles, but their degradation may be compromised to a significant extent, probably not completely blocked as seen in *Δisp6*. In other words, vesicular structures of ABs are likely destroyed, but subsequent degradation of AB contents may be interfered in *Δpqr1* vacuoles. Atg15, the vacuolar lipase for degrading AB membranes, may sustain the function at least partially, even in *Δpqr1* (66). EDAMs could be undigested aggregated cytosolic components derived from ABs. In *Arabidopsis* mutants of *ATG2*, *ATG18a*, and *ATG7*, aggregated peroxisome containing catalase was seen as electron-dense structure similar to EDAM (67). Another interesting possibility is that EDAM is related to hyperaccumulated polyP. As EDAM has no surrounded membrane, EDAM could be a distinct liquid-phase inside vacuole, serving as a space for specific vacuolar functions. Exploring the nature of EDAM will be of great interest.

In spite of the difference in AB accumulation, the similar phenotypes of *Δisp6* and *Δpqr*1, biochemical data obtained using GFP-Atg8 and Cpy1-GFP, and TEM analysis lend credence to the idea that vacuolar degradation, not autophagosome formation, is compromised by the failure of phosphate uptake restriction and subsequent hyperaccumulation of polyP, which results from *pqr1*^*+*^ deletion.

The present study suggests that polyP is involved in autophagy-dependent proteolysis. PolyP is a highly anionic phosphate polymer in which a few to hundreds of phosphate groups are connected in tandem by phosphodiester bonds. polyP is thought to occur in all living things, from bacteria to humans, and serves a variety of physiological functions (10, 12, 68), ranging from phosphate storage and activation of LON protease, which supplies amino acids under starvation and σ-factor regulation in prokaryotes (19, 69), to regulation of blood coagulation and inflammation, stabilization of protein structure, mTOR activity, neural functions, DNA damage responses, energy metabolism, and more, in eukaryotes from yeasts to humans (12, 70, 71, 72, 73). Recently, protein polyphosphorylation has been discovered in budding yeast (74, 75).

While polyP is synthesized in abiotic environmental processes, *e.g.*, volcanic eruptions, polyP is enzymatically synthesized in cells and several polyP synthases have been reported (12). In bacteria, polyphosphate kinases synthesize polyP. The slime mold, *Dictyostelium discoideum*, and some other eukaryotes also possess polyphosphate kinase(s), which probably were also acquired *via* horizontal gene transfer from bacteria (63, 64). In the budding yeast, the VTC complex on vacuolar membranes, composed of Vtc1, Vtc2 or 3, and Vtc4 (the catalytic subunit), synthesizes polyP (46). Another protein, Vtc5, interacts with the VTC complex to activate polyP synthesis (76). *S. pombe* possesses potential orthologs of budding yeast Vtc1, Vtc2, and Vtc4, namely Nrf1, Vtc2, and Vtc4, respectively. There is no apparent counterpart of Vtc5 in *S. pombe*. In metazoa and higher plants, polyphosphate synthases have not been identified.

In the budding yeast, the VTC complex has been intensively studied after the discovery that it is associated with vacuole fusion (77) and that it is apparently involved in several other cellular pathways (46, 78), such as the stability of V-ATPase and microautophagy (79, 80). Our study demonstrated that *Δpqr1* contained 5× more polyP than WT. Deletion of *vtc2*^*+*^ and *vtc4*^*+*^ completely suppressed hyperaccumulation of polyphosphate, viability loss, and defective autophagy-dependent proteolysis seen in *Δpqr1*. Withdrawal of phosphate from the medium also suppressed the phenotypes of *Δpqr1*, including lethality, defective proteolysis, and polyP accumulation, even if subunits of the VTC complex were not mutated. Hence, these results strongly suggest that excessive polyphosphate accumulation causes defective proteolysis, although they do not entirely exclude the possibilities that functions of the VTC complex unrelated to polyP could also be involved to some extent.

Molecular mechanisms by which polyP affects proteolysis in vacuoles will be a central question in a future study. As defects of vacuolar proteolysis could have various causes, *e.g.*, cation chelation, inappropriate pH, membrane fusion defects, aggregation, or unfolding of vacuolar enzymes, protein transport, *etc.*, relationships between these factors and polyP need to be analyzed. PolyP, a typical polyelectrolyte, could form a complex with proteins: protein–polyelectrolyte complex (PPC). Forming such liquid droplet of PPC in vacuoles, polyP could control enzymatic activities of vacuolar proteases. Identification of polyP interacting proteins in *Δpqr1* cells will be of interest in future study. Genetic screening to isolate extragenic suppressor mutants of *Δpqr1* may offer further mechanistic insights. Very recently, it was reported that proteins important for vacuolar functions, Prb1 and Apl5, are targets of lysine polyphosphorylation in *S. cerevisiae* (81). Although the physiological significance of polyphosphorylation of vacuolar proteins has not been addressed, it would be worth examining whether *S. pombe* Isp6, the Prb1 ortholog responsible for autophagic proteolysis could be regulated by polyphosphorylation.

Using *S. pombe* as a model, the present study further illuminated the link between polyP homeostasis and vacuolar proteolysis requisite for completion of starvation-induced autophagy for viability during quiescence (Fig. 8). Considering that autophagy, the ubiquitin pathway, and polyP are likely involved in proteostasis and stress responses, failures of which are strongly relevant to human diseases such as neurodegeneration, the relationship between polyP and autophagy will be important to many research fields if it proves widely conserved among eukaryotes.

## Experimental procedures

### *S. pombe* strains, genetics, media, and culture conditions

All *S. pombe* strains used are listed in Table S1. Complete and synthetic minimal media, YES (YE with five supplements: adenine, uracil, leucine, histidine, and lysine) and EMM2 were employed (82). To induce the quiescent/G_0_ phase, *S. pombe* cells were cultured to log-phase in EMM2 at 26 °C and the medium was changed to EMM2–N (EMM2 without nitrogen) by vacuum filtration, as described previously (83). Cellular concentration in media was measured with a CDA-500 particle counter (Sysmex). To analyze cellular viability, 300 cells were spread on YES plates, incubated for 4 to 6 days at 26 °C, and the number of colonies was counted. Gene deletion and epitope tagging were performed as described previously (84).

### Microscopy and flow cytometer analysis

All images were acquired using an Axiovert 200M fluorescent microscope (Carl-Zeiss). 4', 6-diamino-2-phenylindole (DAPI; Nacalai Tesque) was used to stain cell walls and DNA after fixing cells with glutaraldehyde. To stain vacuolar membranes, FM4-64 (Thermo Fisher Scientific) was used, following a previously described protocol (85). To analyze DNA contents, an SH-800SAP cell sorter (SONY) was used. The procedure for preparing *S. pombe* samples was described previously (86).

### Transmission electron microscopy

After being frozen in liquid propane, *S. pombe* cell samples were freeze-substituted with glutaraldehyde (2%), tannic acid (1%) in ethanol at −80 °C for 48 h. These samples were kept at −20 °C for 2 h and shifted to 4 °C for 2 h. After infiltration with propylene oxide, sample blocks were ultrathin sectioned at 80 nm with a diamond knife, stained with uranyl acetate (2%), and stained secondary with lead stain solution. Samples were observed with a JEM-1400Plus transmission electron microscope (JEOL Ltd) at an acceleration voltage of 100 kV.

### Protein extraction and immunoblotting

For immunoblot analysis, proteins were extracted using the trichloroacetic acid (TCA) method described previously (87), except for phosphate transporters that are difficult to extract, apparently due to their tight interaction with membranes (S. Saitoh, personal communication). To extract phosphate transporters, harvested cells were suspended in chilled, denaturing lysis buffer (20 mM Tris-HCl (pH 8.0), 100 mM sodium-phosphate buffer (pH 8.0), 150 mM NaCl, 1% Triton-X100, 8 M urea, 1 mM phenylmethylsulfonyl fluoride (PMSF)) containing a protease inhibitor cocktail (Nacalai Tesque), and were crushed with glass beads using a Multi-beads shocker (Yasui kikai). Cell extracts were centrifuged at 13,200 rpm for 15 min, and supernatants were boiled at 70 °C for 10 min in the presence of LDS-PAGE sample buffer and 2-mercaptoethanol. Identical amounts of protein were separated on SDS-PAGE gels and transferred to nitrocellulose membranes. Anti-GFP (1/1000, Roche), anti-DYKDDDYK for detecting FLAG tag (1/1000, FUJIFILM Wako), anti-V5 (1/1000, FUJIFILM Wako), anti-Cdc13 (1/1000, Abcam), anti-PSTAIRE (1/1000, SIGMA), anti-ubiquitin (1/1000, 2C5, MBL), anti-phospho-S6 kinase (1/2000, Cell Signaling Technology), and anti-α-Tubulin (TAT1, a gift from Dr Keith Gull) monoclonal antibodies were used as primary antibodies. Except for anti-phospho-S6 kinase, 2% skim milk in phosphate-buffered saline (PBS) was used for membrane blocking and antibody dilution. In the case of anti-phospho-S6 kinase, tris-buffered saline (TBS) was used instead of PBS. Horseradish-peroxidase-conjugated secondary antibodies (Promega) and Clarity Western ECL substrate (BIO-RAD) were used. Chemiluminescent signals were detected with a Lumino-Image Analyzer LAS4000 (GE Healthcare). The software ImageJ was used for quantification.

### Detection of Pho84 ubiquitination

His6-tagged ubiquitin (his6-Ub) or ubiquitin (Ub) was overproduced from plasmids with the inducible nmt1 promoter in WT or *Δpqr1* cells in which the endogenous *pho84*^*+*^ gene was replaced with a *pho84*^*+*^-GFP fusion gene. These cells were cultured in EMM2 without thiamin for 20 h to induce expression of his6-Ub or Ub, and then cells were harvested. Harvested cells (8 × 10^8^ cells) were suspended in 400 μl of denaturing lysis buffer containing 10 mM imidazole (pH 8.0), and proteins were extracted as described above. Protein extracts were incubated with 40 μl of Ni-agarose beads (QIAGEN) at 4 °C for 120 min to purify proteins covalently bonded to His6-Ub. Then Ni-agarose beads were washed 4× with denaturing lysis buffer containing 20 mM imidazole. After washing, Ni-agarose beads were suspended in 1× LDS sample buffer with 2-mercaptoethanol and were boiled at 70 °C for 10 min to solubilize bead-bound proteins.

### Intracellular phosphate and polyphosphate quantification

To quantify intracellular total phosphate, we followed a procedure described previously (88). To purify and quantify intracellular polyphosphate, we followed procedures previously described, with modifications (89, 90). First, intracellular polyphosphate was purified. Harvested cells (5 × 10^7^ cells) were washed carefully 4× with ice-cold water and suspended in 400 μl of Breaking buffer (10 mM Tris-HCl (pH 8.0), 1 mM EDTA (pH 8.0), 100 mM NaCl, 2% Triton-X100, 1% SDS). Then 300 μl of neutral phenol and an appropriate mass of glass beads were added. Cells were crushed with a Multi-Beads shocker. To the cell extract, 300 μl of chloroform was added and mixed, and the aqueous phase was retrieved after centrifugation. After chloroform treatment was repeated once more, the resulting aqueous phase was treated with DNase I and RNase A to remove nucleic acids. Then polyphosphate was purified by ethanol precipitation. To quantify polyphosphate, purified polyphosphate was hydrolyzed in H_2_SO_4_ and the concentration of released phosphate was measured with a Malachite Green Phosphate Assay Kit (R&D Systems, Inc)

## Data availability

All the data described are located in this article.

## Supporting information

This article contains supporting information (34).

## Conflict of interest

The authors declare no conflicts of interest in regard to this article.
