# Supplementary material for: Regulation of inorganic polyphosphate is required for proper vacuolar proteolysis in fission yeast
Source: J Biol Chem. 2021 Jun 18;297(1):100891. doi: 10.1016/j.jbc.2021.100891 (PMC8294586; doi:10.1016/j.jbc.2021.100891)
Supplement: Supplemental Figures S1–S8 and Table S1 [file mmc1.pdf]

Supplementary Information

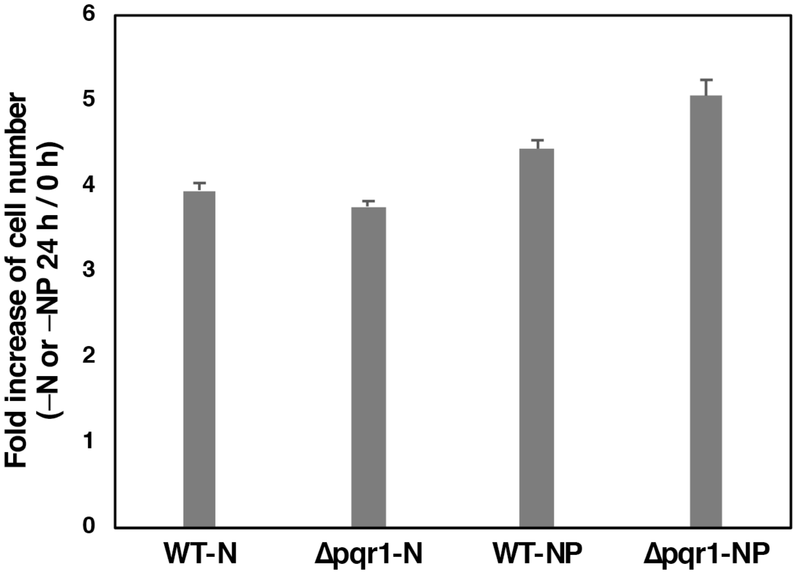

FIG S1. Cell number increases of WT and Δ*pqr1* after medium shift. A. Increases of cell concentrations 24 h after the shift to EMM2–N or EMM2–NP. Both WT and Δ*pqr*1 divided twice; therefore, cell densities increased approximately 4x. Experiments were repeated 3x, and means and SDs are presented.

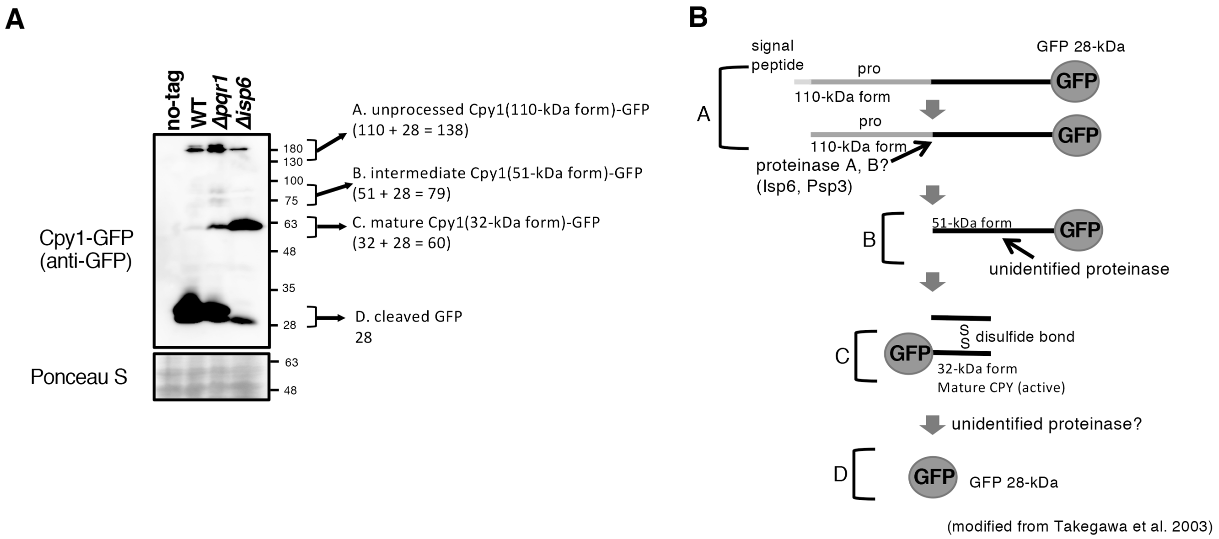

FIG S2. Maturation of vacuolar protease Cpy1 is abnormal in Δ*pqr1*. **A.** Cpy1-GFP maturation was examined by anti-GFP immunoblot in indicated strains. In WT, GFP monomer (D) was abundant as active vacuolar proteases may attack the fusion protein. In Δ*pqr1*, Cpy1-GFP fusion proteins before final maturation (A and B) were more abundant than in WT. Band 'B' was broad or could be a doublet. The reason is unclear, but it may be due to glycosylation of Cpy1 (34). In Δ*isp6*, Cpy1-GFP fusion proteins before final maturation (A and B) were comparable to WT, while GFP monomer (D) was less probable due to insufficient protease activity in Δ*isp6*. **B.** A schematic drawing of Cpy1 maturation in *S. pombe*, modified from original article by Takegawa et al (34).

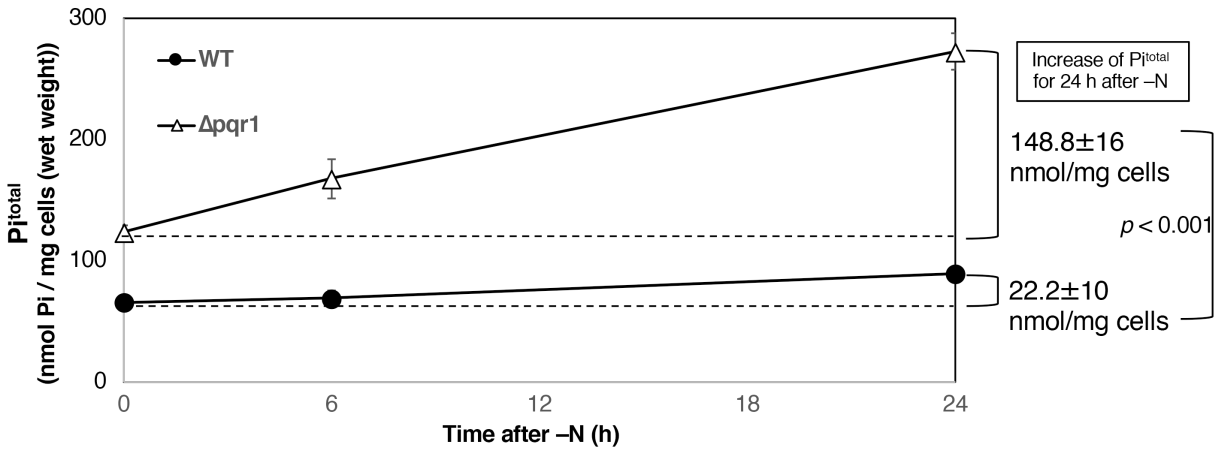

FIG. S3 Time course analysis of Pi^{total} after –N. To compare phosphate uptake in WT and Δ*pqr1*, Pi^{total} of WT and Δ*pqr*1 were quantified at the indicated time after –N. Data were normalized with cell masses (mg) because the cell size is reduced in a time-dependent manner after –N. Experiments were repeated 3x, and means and SDs are presented in the graph. Right: the net increases of Pi^{total} (0 to 24 h) were calculated. Pi^{total} increase in Δ*pqr1* was 144±16 nmol/mg, while 22.2±10 nmol/mg in WT ($p < 0.001$).

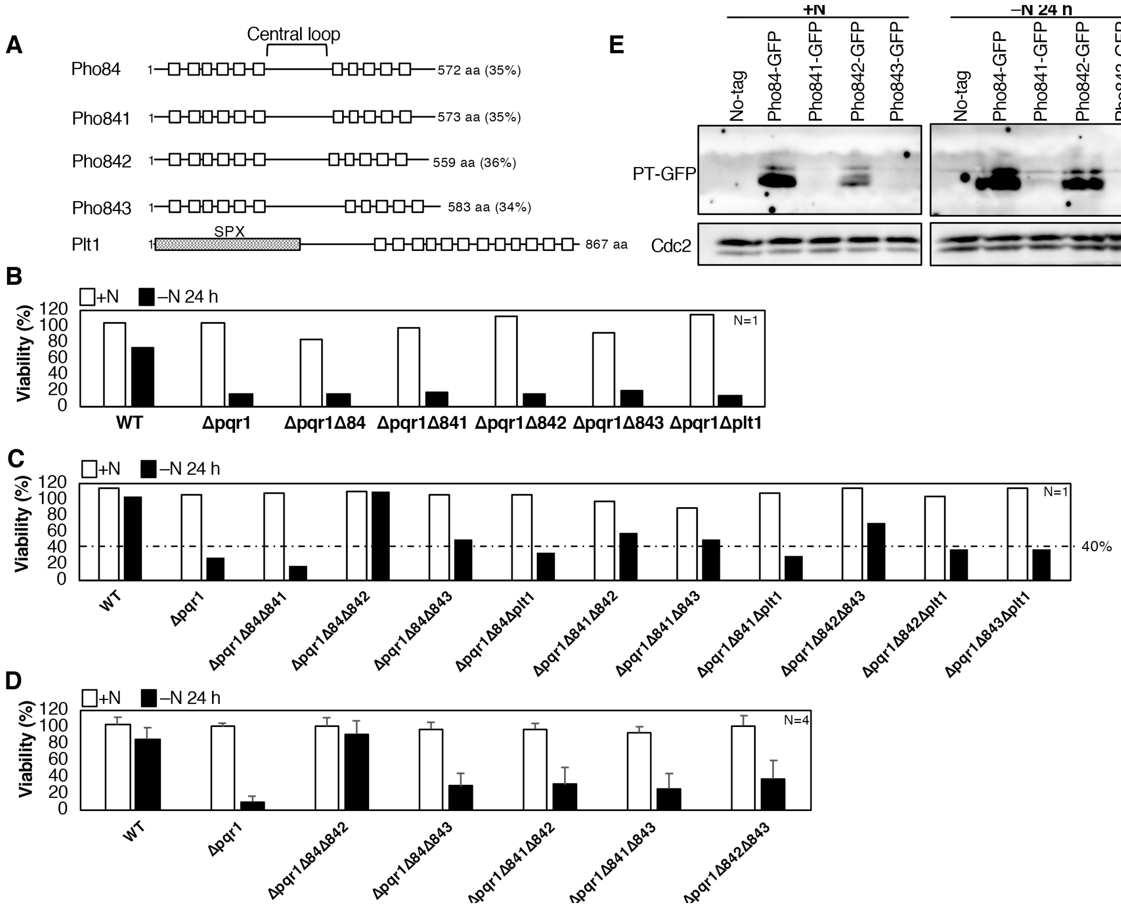

FIG S4. Genetic interactions between Δ*pqr1* and gene deletions of phosphate transporters. A. Schematic drawings of protein structure of five putative phosphate transporters in *S. pombe.* White and gray squares represent transmembrane regions and the SPX domain, respectively. Pho84, 841, 842 and 843 possess long cytoplasmic insertions between the sixth and seventh transmembrane domains (Central loop). **B.** Viabilities of indicated strains are shown. The viability loss of Δ*pqr1* in –N was not affected by single gene deletions of phosphate transporters. The experiment was performed once. **C.** Double gene deletions of phosphate transporters were combined with Δ*pqr1* and viabilities were measured. Δ*pho84*Δ*pho842*, Δ*pho841*Δ*pho842*, Δ*pho84*Δ*pho843* and Δ*pho842*Δ*pho843* seemed to restore viability of Δ*pqr1* to more than 40% (hashed line). The experiment was performed once. **D.** The experiment shown in C was repeated 4x and means and SDs are presented. **E.** Protein concentrations of four high-affinity phosphate transporters were examined by immunoblot. Each endogenous phosphate transporter gene was replaced with a GFP-fused gene, all of which were controlled by their own promoters. Under ±N conditions, Pho84-GFP was the most abundant and Pho842-GFP was next most. Pho841 and Pho843 were hardly detectable, while Pho841 seemed to increase a little after –N.

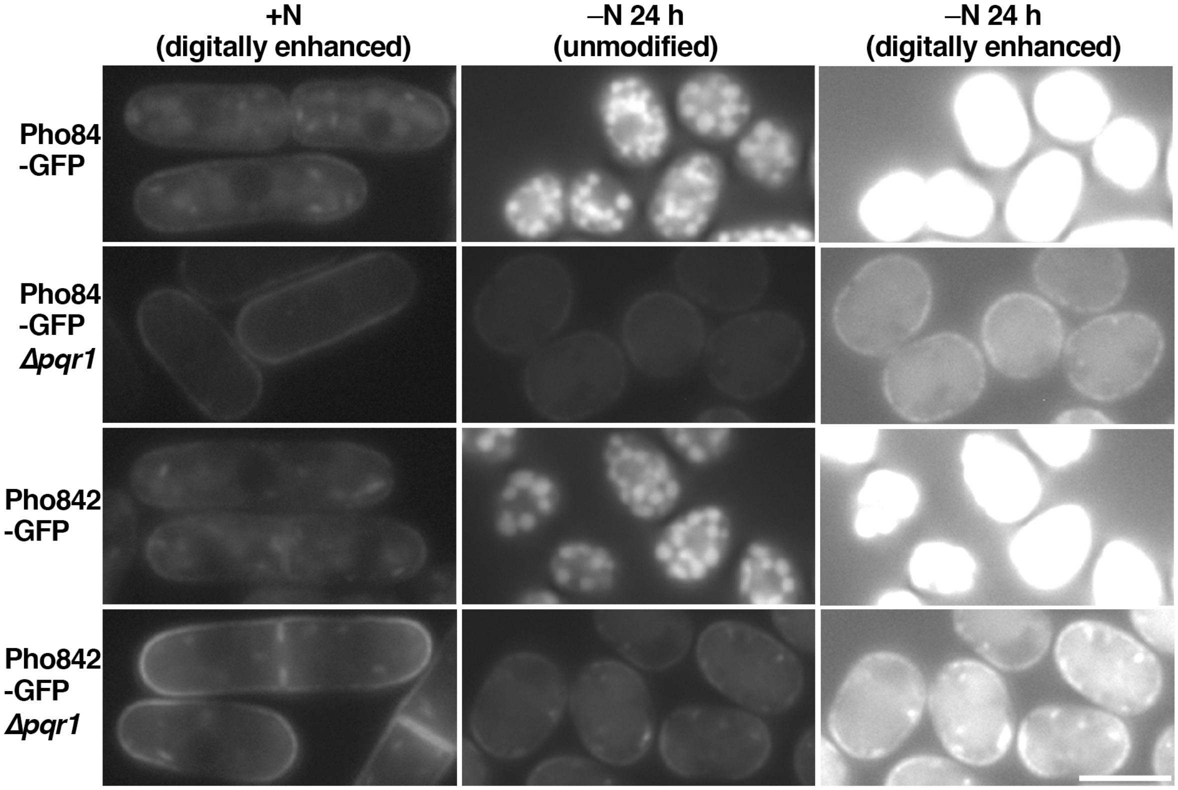

FIG. S5 Localization of Pho84-GFP and Pho842-GFP. Signal levels of Pho84-GFP and Pho842-GFP were elevated drastically in WT 24 h after –N and were difficult to compare with signals obtained under other conditions. Therefore, data in Figure 5 were digitally modified using Photoshop level adjustment and presented as described in the text. Here, all images are shown. The left column presents images obtained from cells under +N conditions, and fluorescent signals were digitally enhanced. The central column presents images from cells under –N conditions and no digital modifications were made. The right column presents digitally enhanced versions of the central images. Digital modifications to the left and right columns were the same. Bar, 5 µm.

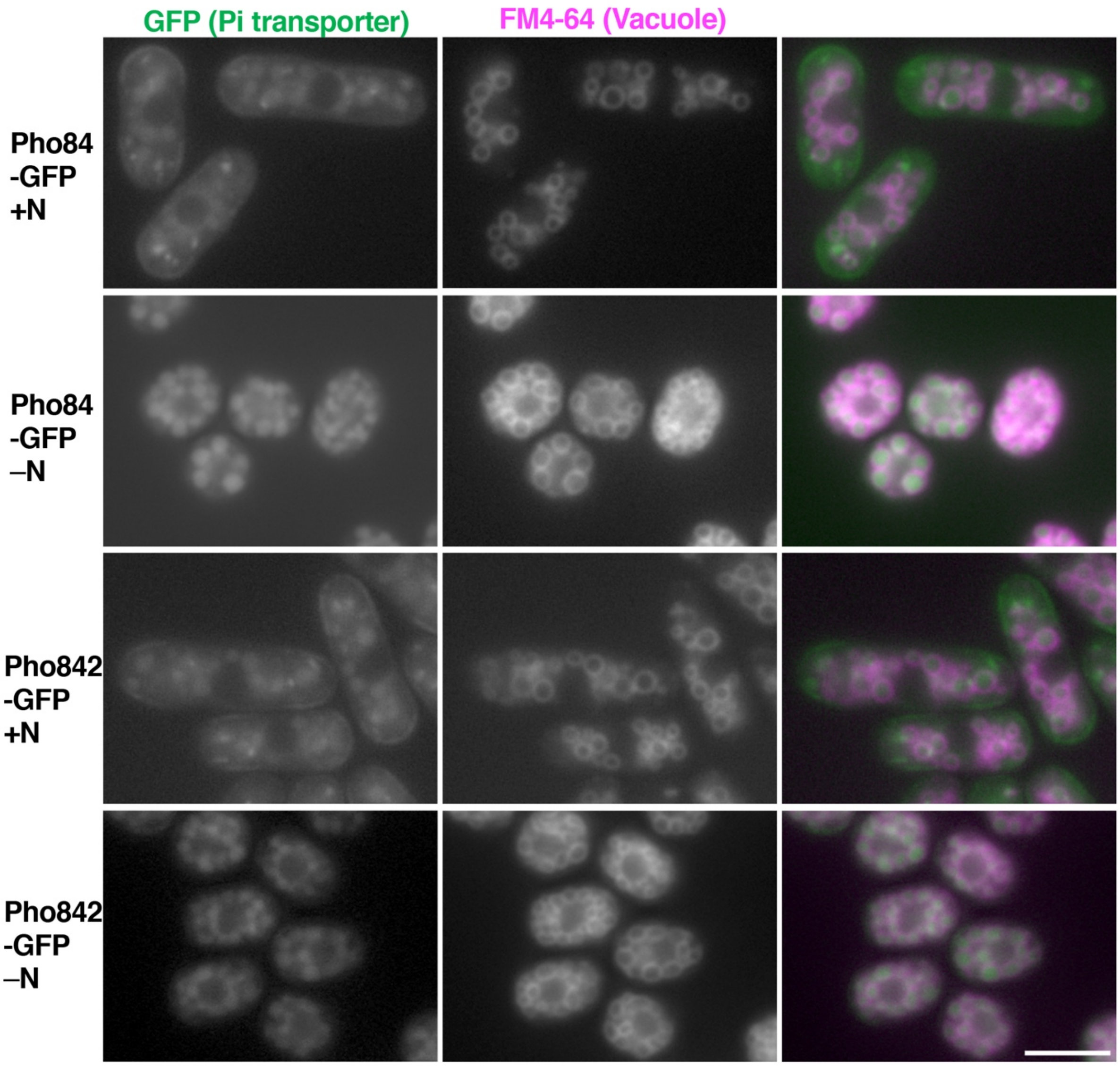

FIG. S6 Co-localization of phosphate transporters and vacuoles. Pho84 and Pho842 were visualized with GFP (green) and vacuolar membranes were stained with FM4-64 (magenta). Typically, in –N, GFP signals were surrounded by FM4-64 signals, suggesting that phosphate transporters were translocated to vacuoles. Bar, 5 µm.

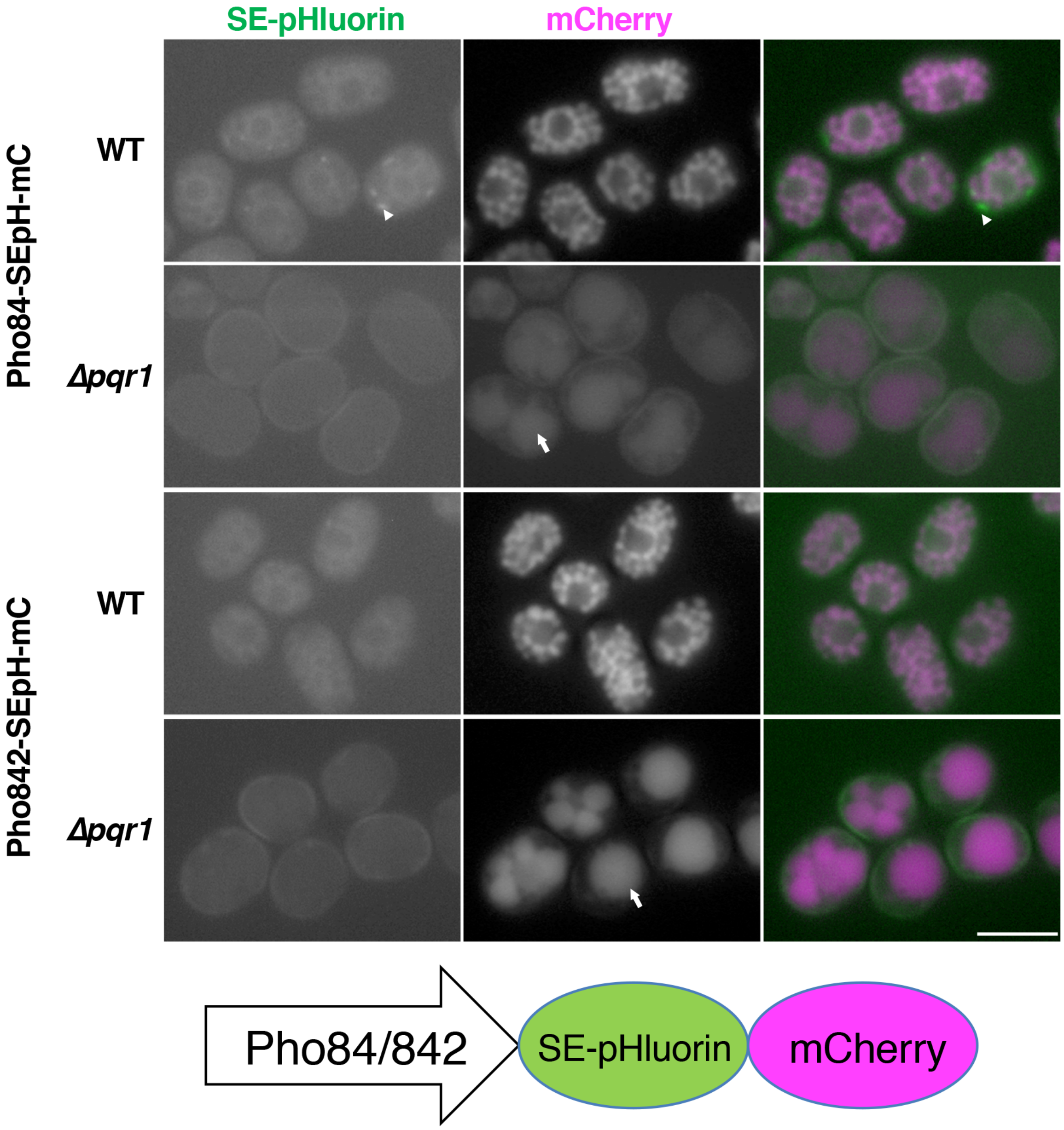

FIG. S7 Pho84 and Pho842 remained on the plasma membrane in Δ*pqr1* after –N. To reduce halation from hyper GFP signal in vacuoles, a new tandem tag, Super-Ecliptic-pHluorin (SEpH, green) - mCherry (magenta) was fused chromosomally to Pho84 and Pho842. SEpH is hyper-sensitive to low pH and mCherry is less affected by pH. Clearly, both Pho84-SEpH and Pho842-SEpH were brighter on the plasma membrane in Δ*pqr1* than in WT. Pho84-mCherry and Pho842-mCherry were clearly seen in vacuole like structures in Δ*pqr1* (arrows), but darker than in WT. Bar,5 µm. Bottom; schematic drawing of fusion proteins.

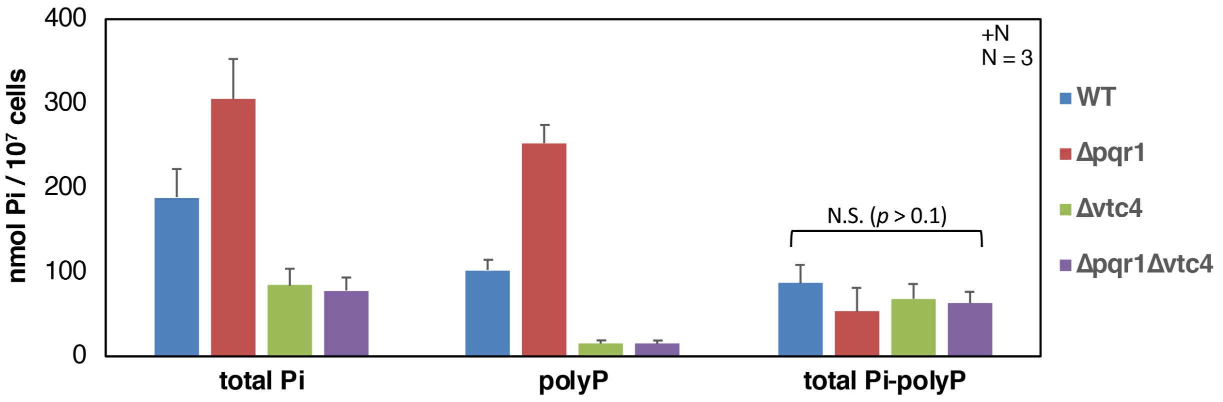

FIG. S8 Comparison of amounts of Pi^{total} and polyP. Pi^{total} and polyP were quantified in indicated strains under +N conditions. Non-polyP phosphate in the cell was calculated and shown as 'Pi^{total} – polyP', and was not significantly different among strains ($p > 0.1$). Experiments were repeated 3x, and means and SDs are presented.

Table S1 The list of *S. pombe* strains used in this study

Strain	Genotype	Source
KP162	h^- Δ*pqr1*::kanMX	BIONEER[#]
KP445	h^- Δ*atg3*::kanMX	BIONEER[#]
KP446	h^- Δ*atg5*::kanMX	BIONEER[#]
KP447	h^- Δ*atg6*::kanMX	BIONEER[#]
KP448	h^- Δ*atg7*::kanMX	BIONEER[#]
KP449	h^- Δ*atg13*::kanMX	BIONEER[#]
KP570	h^+ Δ*pqr1*::natMX Δ*atg8*::kanMX	This study[$]
KP573	h^- Δ*pqr1*::hphMX	This study
KP576	h^- Δ*pqr1*::natMX Δ*atg5*::kanMX	This study[$]
KP580	h^- Δ*pqr1*::natMX Δ*atg3*::kanMX	This study[$]
KP582	h^- Δ*pqr1*::natMX Δ*atg7*::kanMX	This study[$]
KP584	h^- Δ*pqr1*::natMX Δ*atg6*::kanMX	This study[$]
KP586	h^- Δ*pqr1*::natMX Δ*atg13*::kanMX	This study[$]
KP596	h^+ Δ*atg3*::*kanMX GFP-atg8*$^+$:*leu1*$^+$ *leu1-32*	This study[$]
KP598	h^+ Δ*atg5*::*kanMX GFP-atg8*$^+$:*leu1*$^+$ *leu1-32*	This study[$]
KP600	h^+ Δ*atg13*::*kanMX GFP-atg8*$^+$:*leu1*$^+$ *leu1-32*	This study[$]
KP622	h^- mCherry-*atg8*$^+$:*leu1*$^+$ *leu1-32*	This study
KP623	h^- Δ*pqr1*::hphMX mCherry-*atg8*$^+$:*leu1*$^+$ *leu1-32*	This study
KP662	h^- *atg14*$^+$-*GFP*::kanMX	This study
KP663	h^- *atg16*$^+$-GFP::kanMX	This study
KP665	h^+ Δ*pqr1*:: hphMX *atg14*$^+$-GFP::kanMX	This study
KP666	h^+ Δ*pqr1*:: hphMX *atg16*$^+$-GFP::kanMX	This study
KP96	h^- Δ*atg8*::hphMX	This study
SN128	h^- *pho84*$^+$-GFP::kanMX	This study
SN138	h^- *pho842*$^+$-GFP::kanMX	This study
SN172	h^- Δ*vtc2*::kanMX	This study
SN178	h^- Δ*vtc4*::kanMX	This study
SN194	h^- *pho84*$^+$-GFP::kanMX Δ*pqr1*::hphMX	This study
SN198	h^- *pho842*$^+$-GFP::kanMX Δ*pqr1*::hphMX	This study
SN204	h^- Δ*vtc2*::kanMX Δ*pqr1*::hphMX	This study
SN207	h^- Δ*vtc4*::kanMX Δ*pqr1*::hphMX	This study

SN210	h^+ Δ*pqr1*::hphMX Δ*pho84*::natMX Δ*pho842*::kanMX	This study
SN230	h^- Δ*pho84*::natMX Δ*pho842*::kanMX	This study
SN259	h^- Δ*vtc2*::kanMX Δ*pqr1*::hphMX *GFP-atg8*$^+$:*leu1*$^+$ *leu1-32*	This study
SN263	h^- Δ*vtc2*::kanMX *GFP-atg8*$^+$:*leu1*$^+$ *leu1-32*	This study
SN266	h^- Δ*vtc4*::kanMX Δ*pqr*1::hphMX *GFP-atg8*$^+$:*leu1*$^+$ *leu1-32*	This study
SN270	h^- Δ*vtc2*::kanMX *GFP-atg8*$^+$:*leu1*$^+$ *leu1-32*	This study
SN274	h^- Δ*pqr1*::hphMX Δ*pho84*::natMX Δ*pho842*::kanMX *GFP-atg8*$^+$:*leu1*$^+$ *leu1-32*	This study
SN286	h^- Δ*pho84*::natMX Δ*pho842*::kanMX *GFP-atg8*$^+$:*leu1*$^+$ *leu1-32*	This study
TK375	h^+ *GFP-atg8*$^+$:*leu1*$^+$ *leu1-32*	This study
TK427	h^- Δ*pqr1::kanMX GFP-atg8*$^+$:*leu1*$^+$ *leu1-32*	This study
TKN33	h^- *atg13*$^+$-V5:natMX *psk1*$^+$-FLAG5:kanMX	This study*
TKN40	h^- Δ*pqr1*::hphMX *atg13*$^+$-V5:natMX *psk1*$^+$-FLAG5:kanMX	This study*
WT(972h^-)	h^-	NBRP yeast

*: *psk1*$^+$-FLAG5::kanMX was derived from NBRP yeast Japan as FY31941 (h^- *leu1-32 psk1*$^+$-FLAG5:kanMX). #: These gene-deletion mutants were originally derived from haploid gene deletion library (BIONEER). They were back-crossed with WT to remove auxotrophic mutations.
$: including gene-deletion derived from BIONEER haploid gene deletion library
